# Comprehensive characterization of high-risk coding and non-coding single nucleotide polymorphisms of human CXCR4 gene

**DOI:** 10.1371/journal.pone.0312733

**Published:** 2024-12-23

**Authors:** Bonoshree Sarkar, Muhammad Safiul Alam Mondal, Taibur Rahman, Md. Ismail Hosen, Atiqur Rahman

**Affiliations:** 1 Infection Biology Laboratory, Department of Biochemistry and Molecular Biology, University of Dhaka, Dhaka, Bangladesh; 2 Clinical Biochemistry and Translational Medicine Laboratory, Department of Biochemistry and Molecular Biology, University of Dhaka, Dhaka, Bangladesh; CSIR-IHBT: Institute of Himalayan Bioresource Technology CSIR, INDIA

## Abstract

CXCR4, a chemokine receptor known as Fusin or CD184, spans the outer membrane of various human cells, including leukocytes. This receptor is essential for HIV infection as well as for many vital cellular processes and is implicated to be associated with multiple pathologies, including cancers. This study employs various computational tools to investigate the molecular effects of disease-vulnerable germ-line missense and non-coding SNPs of the CXCR4 gene. In this investigation, the tools SIFT, PROVEAN, PolyPhen-2, PANTHER, SNAP 2.0, PhD-SNP, and SNPs&GO were used to predict potentially harmful and disease-causing nsSNPs in CXCR4. Additionally, their impact on protein stability was examined by I-mutant 3.0, MUpro, Consurf, and Netsurf 2.0, combined with conservation and solvent accessibility analyses. Structural analysis with normal and mutant residues of the protein harboring these disease-associated functional SNPs was conducted using TM-align and SWIS MODEL, with visualization aided by PyMOL and the BIOVINA Discovery Studio Visualizer. The functional impact of wild-type and mutated CXCR4 variants was evaluated through molecular docking with its natural ligand CXCR4-modulator 1, using the PyRx tool. Non-coding SNPs in the 3′ -UTR were investigated for their regulatory effects on miRNA binding sites using PolymiRTS. Five non-coding SNPs were identified in the 3′-UTR that can disrupt existing miRNA binding sites or create new ones. Non-coding SNPs in the 5′ and 3′-UTRs, as well as in intronic regions, were also examined for their potential roles in gene expression regulation. Furthermore, RegulomeDB databases were employed to assess the regulatory potential of these non-coding SNPs based on chromatin state and protein binding regulation. In the mostly annotated variant (ENSP00000241393) of the CXCR4 gene, we found 23 highly deleterious and pathogenic nsSNPs and these were selected for in-depth analysis. Among the 23 nsSNPs, five (G55V, H79P, L80P, H113P, and P299L) displayed notable structural alternation, with elevated RMSD values and reduced TM (TM-score) values. A molecular docking study revealed the significant impact of the H113P variant on the protein-ligand binding affinity, supported by MD simulation over 100 nanoseconds, which highlighted substantial stability differences between wild-type and H113P mutated proteins during ligand binding. This comprehensive analysis shed light on the potential functional consequences of genetic variation in the CXCR genes, offering valuable insights into the implications of disease susceptibility and may pave the way for future therapeutic interventions.

## Introduction

Single Nucleotide Polymorphisms (SNPs) are a critical aspect of human genetics, exerting a profound effect on various aspects of biology. They are of particular importance because the coding region of human DNA contains approximately 500,000 SNPs, which can directly or indirectly influence the structure and function of the corresponding proteins [1, 2]. The majority of disease-associated SNPs discovered by genome-wide association studies (GWAS) are mostly located in the non-coding regions of the genome [3, 4]. Nevertheless, both post-transcriptional and post-translational events can be affected by mutations in these regions [5, 6]. Moreover, SNPs in UTRs (Untranslated regions) can modulate protein expression and RNA binding functionalities [7, 8]. These SNP-induced alterations, ranging from changes in charge and shape to functional perturbations, possess the potential to disrupt cellular and physiological homeostasis [9, 10].

Among the myriads of genes subject to SNP-mediated modulation, the CXCR4 (C-X-C motif chemokine receptor 4) gene emerges as a focal point of interest. Recognized alternatively as Fusin or CD184, CXCR4 is a type of α-chemokine receptor that spans the outer membrane of many cell types, including lymphocytes, hematopoietic stem cells, epithelial cells, endothelial cells, and cancer cells [11–13]. The gene, located at 2q21, encodes a protein comprising 352 amino acids (MW 48KDa). Notably, it exhibits a few isoforms arising from alternative splicing events [14]. The protein catches the most attention due to its potent role in the entry of HIV into human lymphocytes. T-cell trophic HIV viruses use CXCR4 as the co-receptor for infection [12, 15]. However, the receptor plays key roles in several other cellular processes such as cell migration, hematopoiesis, cell homing, and proliferation of non-hematopoietic cells [16].

Several clinical significances of the receptor have already been established. Mutations in this gene are responsible for WHIM (warts, hypogammaglobulinemia, infections, and myelokathexis) syndrome [17]. The receptor is overexpressed in breast cancer cells [18], and its upregulation is also observed during the hormone replacement therapy cycles in the endometrium [19]. In addition to its role in breast cancer, the receptor has also been found to be expressed in ovarian cancer, prostate cancer, melanoma, and some other cancers, while its expression is generally very low to absent in normal healthy adult cells [17]. Moreover, correlations between CXCR4 gene expression and metastasis have been unraveled in lung, liver, and bone marrow cancers. Notably, mutations in the CXCR4 gene have been implicated in aberrant neuronal distribution, suggesting potential links to epilepsy [20].

Mutations in the CXCR4 gene hold the potential to lead to serious outcomes, resulting in disturbances in protein expression, function, and regulation, all of which are capable of rendering diverse consequences on the overall well-being of a person. As a receptor, a mutation in this gene can cause dysregulation in its ligand binding, which can trigger severe downstream effects. The most established ligand of the receptor is CXCL12, also known as SDF-1 (stromal-derived factor-1) [15, 21, 22]. Yet, interaction with other molecules such as ubiquitin and USP14 has also been studied [23]. The receptor undergoes dynamic dimerization and the receptor-ligand interaction usually occurs in an autocrine fashion [24].

Despite playing so many key physiological roles, SNPs in this protein and their potential effects have never been extensively analyzed before. SNPs in this protein can alter many regular physiological processes as well as change the way certain viruses such as HIV attack humans. The binding of the natural ligand of the protein or any drug that is designed to target this molecule can also exhibit various efficacy depending on the SNP profile [25] within this gene. Our study aimed to address this knowledge gap by investigating the potential deleterious and pathogenic effects of SNPs in the protein. Using various computational tools and databases, the change in the structure and function of the protein rendered by the SNPs, as well as the clinical relevance of the genetic variance was assessed [26, 27].

It was anticipated in this study that deleterious and disease-causing nsSNPs would have an impact on the structure of the CXCR4 protein. A parallel analysis was found to be noted previously on the CCR5 gene (C-C chemokine receptor type five), which is critical for the entry of macrophage-tropic HIV strains [28]. While CCR5 serves as the initial receptor for HIV infection, CXCR4 becomes the primary co-receptor as the virus progresses to AIDS. Notably, the CCR5Δ32 mutation provides resistance to HIV infection by altering the receptor’s structure and function [29, 30]. Similarly, certain SNPs in CCR5 (e.g., rs145061115, rs199824195, and rs201797884) have been associated with resistance to HIV by affecting receptor stability and ligand binding [31].

To investigate the effect on the CXCR4 receptor, along with finding the effect of SNPs on the structure, function, and stability, we measured the binding affinity of the mutated protein against its synthetic ligand, CXCR4-modulator-1 (PubChem CID 92416899), and compared it to the wild-type protein using molecular docking [32]. Along with molecular docking, Molecular Dynamics (MD) simulation studies were used to obtain the dynamic expression and to comprehend the stability of the protein-ligand complex following docking [33].

Overall, the findings from this study can advance our knowledge of the genetic aspects of the CXCR4 gene. These insights can be applied to comprehend the molecular basis of relevant diseases and to design novel therapeutics, thereby contributing to improved clinical outcomes.

## Materials and methods

**Fig 1:**
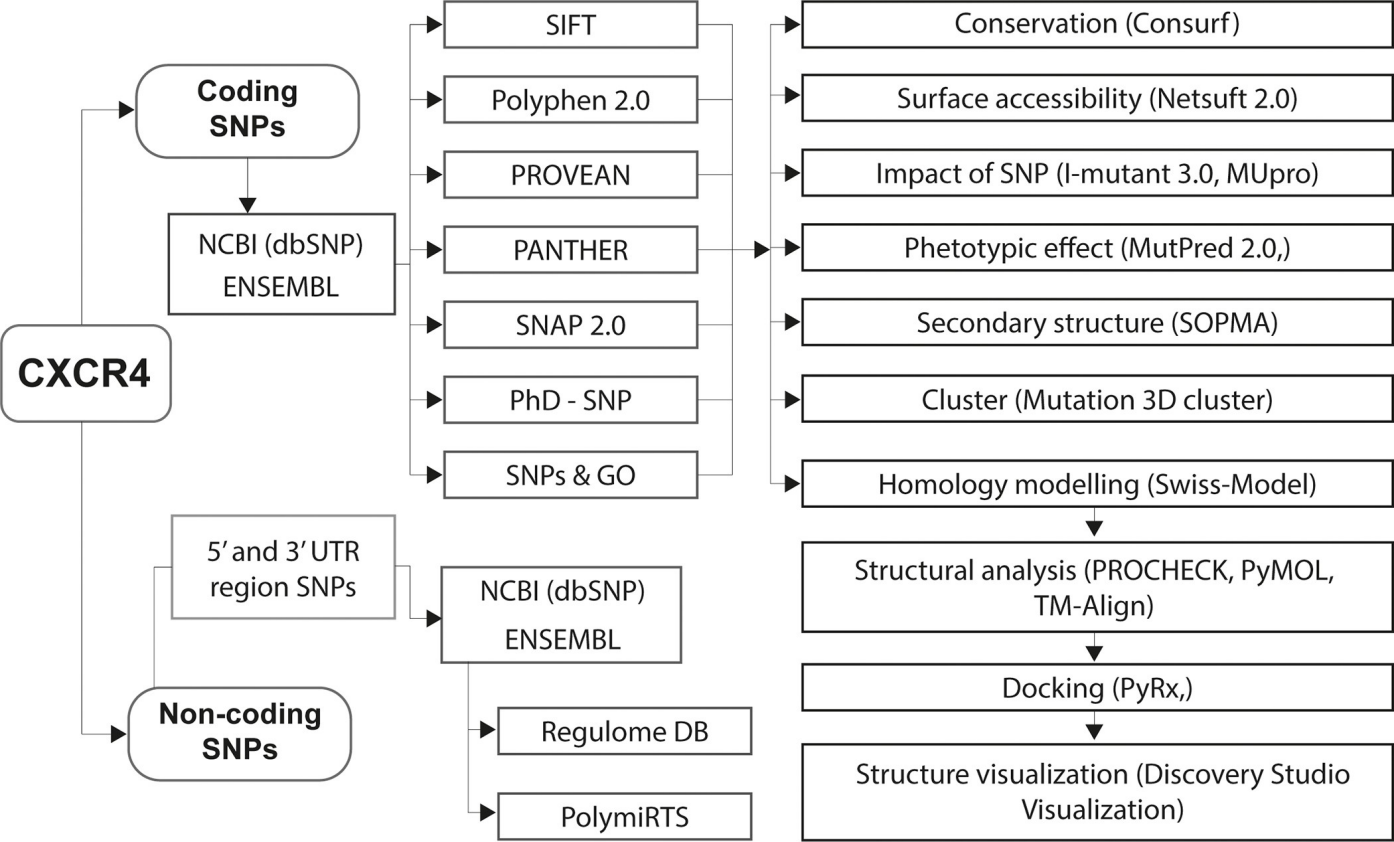
Flowchart of the methodology used in this study. All the coding SNPs obtained from the NCBI and ENSEMBL databases were filtered to find out the most deleterious and disease-causing SNPs using seven different bioinformatics tools. Then the filtered nsSNPs were assessed for their effect on the secondary and tertiary structure of the protein. The effect of a drug was predicted against nsSNPs that have a massive effect on the protein structure. The consequences of the non-coding SNPs were also investigated.

The major steps followed in this study are presented in Fig 1.

### Retrieval of non-synonymous Single Nucleotide Polymorphisms (nsSNPs)

The nsSNPs of the human CXCR4 gene were retrieved from the dbSNP of NCBI (National Center for Biotechnology Information) [34]. Missense SNPs from protein ID ENSP00000241393, ENSP00000440311, and ENSP00000386884 were collected for further computational processes and analyses. The SNPs of UTR regions were also retrieved.

### Prediction of deleterious SNPs

For predicting the most deleterious SNPs from the retrieved nsSNPs 7 bioinformatics tools were used–SIFT (Sorting Intolerant From Tolerant) [35], Polyphen 2.0 (Polymorphism Phenotyping v2) [36], PROVEAN (Protein Variation Effect Analyzer) [37], PANTHER (position-specific evolutionary preservation) [38], SNAP 2.0 (Screening for Non-Acceptable Polymorphism v2) [39], PhD–SNP (Predictor of human Deleterious Single Nucleotide Polymorphisms) [40], SNPs & GO [41]. Among these, SIFT, Polyphen 2.0, PROVEAN, and PANTHER predict how the SNPs would affect the protein’s structure and function. rsID of the protein and SNPs were used as input of SIFT. A prediction score of <0.05 is considered deleterious and ≥0.05 is considered tolerated. In the Polyphen 2.0 tool, the FASTA format of the protein was used as an input with the amino acid substitution. Position Specific Independent Count (PSIC) >0.85 is considered as ‘Possibly damaging’, ~1 is considered as ‘Probably damaging’, and <0.85 is ‘Benign’. It provides results in two sections–HumDiv and HumVar, where HumDiv is related to evolutionary conservation and HumVar is related to human mendelian disease variants. Like Polyphen 2.0, PROVEAN also takes the FASTA format of protein as input, and score ≤-2.5 defines deleterious SNPs, and >2.5 defines tolerated SNPs. But in PANTHER basic amino acid sequences with amino acid variants were used as input and the output is the estimated period (in millions of years) for preserving a specific amino acid. The longer the preservation time, the amino acid variants become more deleterious. The highest preservation time 1036 million years was observed. Like the previous tools, SNAP 2.0 also takes the FASTA format of the protein sequence as an input and using a machine learning device named Neutral Network predicts the effect of the mutations. The threshold value of this tool: for neutral: -100 ≤ SNAP2 score≤ 0, and for effect: 0< SNAP2 score≤ 100.

Two other tools, PhD-SNP and SNP & GO, were employed to predict the disease-causing effects of SNPs. PhD-SNP assesses whether mutations in nsSNPs within a human gene can lead to disease or have no significant effect, requiring protein sequence and amino acid variations as input. In contrast, SNP & GO only requires a UniProt Accession number along with amino acid variation and position number to predict the disease-causing potential of SNPs.

From the nsSNPs retrieved from the NCBI database, those identified as both deleterious and disease-causing by all seven tools were selected for further investigation.

### Predicting the effects of SNPs on the stability of protein

I-Mutant 3.0 and MUpro tools were used for predicting the effects of SNPs on protein stability. I-Mutant 3.0 (http://gpcr2.biocomp.unibo.it/cgi/predictors/I-Mutant3.0/I-Mutant3.0.cgi) can predict the stability of a protein with altered residues and provides a numerical estimation of changed free energy by calculating the difference of unfolding Gibbs free energy between the wild type and mutated protein. It is a support vector Machine-based (SVM) predictor. The value is given in kcal/mol units and according to binary classification, DDG > 0 indicates increased protein stability and DDG < 0 means decreased protein stability [42]. This tool was used with default values of other parameters such as temperature fixed at 25˚C and pH was 7.0. The MUpro (http://mupro.proteomics.ics.uci.edu/) tool also provides prediction using SVM along with a large mutation dataset and neural networks. Like the previous, it gives a prediction value of DDG but with a confidence score between– 1 and 1, where DDG < 0 indicates increased stability and DDG > 0 corresponds to decreased stability [43].

### Prediction of sequence conservation and surface accessibility of protein

To predict the conservancy of the amino acids at the SNP positions, a web-based tool Consurf (https://consurf.tau.ac.il/) was utilized. This tool uses CSI-BLAST, PSI-BLAST, or BLAST among homologous sequences to analyze the evolutionary dynamics of the substituted amino acids, creates a phylogenetic tree, and provides a conservation score that is position-specific, on the scale of 1 to 9 (9 refers to the most conserved region) [44]. However, in this tool highly conserved and exposed residues are considered as ‘functional residues’, and highly conserved and buried residues are considered as ‘structural residues’ [45]. All the default parameters, including the UNIREF-90 protein database HMMER homolog search algorithm, and 0.0001 HMMER E-value, were used. After that, for predicting the surface accessibility of the substituted amino acids NetSurfP-2.0 (http://www.cbs.dtu.dk/services/NetSurfP/) was used. Besides, the tool predicts secondary structure and structural disorder of amino acids using conventional long short-term memory neural network-based architecture. The output is given as a Relative Surface Accessibility (RSA) value, where RSA > 25% is considered as exposed and RSA < 25% is considered as buried [46].

### Prediction of post-translational modification (PTM) change

For predicting post-translational modification (PTM) sites, a web-based tool MusiteDeep (https://www.musite.net/) was used. This tool can also provide an interacting review of the predicted PTM sites in the context of known PTM annotations from UniProt/Swiss-Prot and protein 3D structures through homology-based search. It can predict 13 different modifications [47]; for our protein, all the modifications were predicted using different probability scores.

### Prediction of phenotypic effects

For analyzing phenotypic effects 2 different web tools were used—MutPred2 and HOPE. MutPred2 (http://mutpred.mutdb.org/index.html) contains a pathogenic and unlabeled variants dataset and predicts the alteration in activity and binding of the mutated proteins. It provides a p-value ranging from 0 to 1 depicting clinically significant variants, where scores closer to 1 indicate more pathogenic [48]. HOPE (https://www3.cmbi.umcn.nl/hope/) analyses the effects of SNPs on the 3D structure of proteins by using PDB and UniProt databases. It creates sequence alignment, side-chain modeling, loop building, and energy minimization to predict the effects of SNPs on residue size, hydrophobicity, charge, spatial structure, function, bond difference, etc. [49].

### Structural analysis

Secondary structures, such as α helix, β turn, and coil, were predicted by SOPMA (https://npsa-prabi.ibcp.fr/cgi-bin/npsa_automat.pl?page=/NPSA/npsa_sopma.html). Parameters were set as suggested (17 window lengths, 8 similarity thresholds, and 4 number of states) [50].

To find out the functional domain of the protein on which the SNPs are positioned, the Mutation3D (http://mutation3d.org/index.shtml) tool was utilized. This tool predicts the spatial arrangement of the substituted amino acid residues in proteins and using cluster-based procedure it identifies a cluster of mutated amino acid residues that affect the protein structure most [51].

Homology models were generated using an online homology modeling tool SWISS-MODEL (https://swissmodel.expasy.org/) for the wild type and significant deleterious missense SNPs found in CXCR4. SWISS-MODEL uses a template structure and aligns the target sequence with the template sequence to build the target structure. To predict the structure, the tool uses a PDB template and takes an amino acid sequence as input [52]. The protein sequences of CXCR4 were retrieved from Ensemble.

The most harmful SNPs filtered from the previous analyses were studied in the following tools–SWISS-MODEL structure assessment (https://swissmodel.expasy.org/assess), ERRAT (https://servicesn.mbi.ucla.edu/ERRAT/), and PROCHECK (https://servicesn.mbi.ucla.edu/PROCHECK/). These tools give Ramachandran plot results, MolProbity scores, QMEAN Z-Scores, ERRAT, and PROCHECK scores. All the given scores evaluate the quality of the predicted structures.

Following building the models of wild and mutated proteins of CXCR4, the structures were then analyzed in TM-Align and PyMOL tools. TM-Align (https://zhanglab.ccmb.med.umich.edu/TM-align/) is a web-based tool that compares the protein structure in a sequence-independent manner [53]. PyMOL is a graphic tool that is used for 3D visualization of macromolecules. Both of the tools provide template modeling (TM)- score and root mean square deviation (RMSD)- value. TM-score ranged from 0 to 1, where value from 0.0 to 0.30 indicates random structural similarity, and from 0.5 to 1.00 refers to a perfect match between the wild type and mutated type protein structures [53]. However, in the case of RMSD value, greater values indicate greater structural differences between the wild and mutated types of protein.

To visualize the predicted structure, PyMOL as well as Discovery Studio were used. With PyMOL the distance of amino acids, angles with adjacent amino acids, etc., were measured, while the visualization of the H-bonds and other types of interactions were performed using the Discovery studio. All the outputs help to analyze the effects of SNPs on the structure of our protein of interest.

### Identification of non-coding SNPs of CXCR4 gene

The SNPs of the CXCR4 gene in non-coding regions, especially in the UTR regions, were retrieved from the Ensembl genome browser dataset using specific filters. The functional impacts of these SNPs were analyzed by the RegulomeDB (https://regulomedb.org/regulome-search/) tool. It gives scores in a range between 0 and 1, where scores near 1 indicate more regulatory variants. This tool uses different databases including ENCODE ChIP-seq, FAIRE, DNase I hypersensitive site, eQTL, dsQTL, and ChIP-exo data, and categorizes them in different ranks. These ranks of the SNP provide information about the supporting data of their ability to affect transcription factor binding, gene expression regulation for a protein, alternative binding, chromatin accessibility, etc. [54].

### Prediction of the non-coding SNPs on microRNAs and target sites

PolymiRTS Database 3.0 (https://compbio.uthsc.edu/miRSNP/) was utilized for filtering the SNPs on the non-coding regions of the CXCR4 gene that affect the miRNA seed region and their target sites, Polymorphisms in microRNAs and their target sites. This database uses data from CLASH experiments or cross-linking, ligation, and sequencing of hybrid experiments for predicting the outputs. It also provides the rsID and miR ID of the miRNAs that are affected by selected SNPs. By using the IDs, the miRNAs can be studied in detail [55]. For the non-coding SNPs of CXCR4 genes, only the SNPs on the 3’-UTR regions were considered in this analysis.

### Docking

To predict the effect of the SNPs in the CXCR4 structure, a ligand of the protein, called CXCR4-modulator 1 (PubChem CID 92416899) was docked with the predicted wild type and mutated protein structures using PyRx. Any change in ligand binding ability in the mutated variants compared to that of the wild type was calculated One Way ANOVA.

### Molecular dynamic simulation

A 100 nanosecond molecular dynamics simulation was conducted with GROMACS (version 2020.6) [56]. The CHARMM36m force field was employed for this simulation, and a water-box with edges positioned 1 nanometer away from the protein surface was created using the TIP3 water model. To neutralize the systems, appropriate ions were added. The simulation, with a time step of 2 femtoseconds, employed periodic boundary conditions following energy minimization, isothermal-isochoric (NVT) equilibration, and isobaric (NPT) equilibration of the system. Snapshot intervals were set at 100 picoseconds for trajectory data analysis. After the simulation, the root mean square deviation (RMSD), root mean square fluctuation (RMSF), radius of gyration (RG), and solvent accessible surface area (SASA) analyses were performed using the integrated RMS, RMSF, Gyrate, and SASA modules within GROMACS software. Plots for these analyses were generated using the ggplot2 program in RStudio. High-performance simulation stations with the Ubuntu 20.04.4 LTS operating system were utilized for all MD simulations.

## Results

### Retrieval of non-synonymous SNPs of CXCR4

Polymorphism data for the CXCR4 gene were retrieved from NCBI dbSNP and Ensembl databases. For the CXCR4 protein, a total of 2,870 SNPs was retrieved from the NCBI dbSNP database, which corresponds to r 3 Ensembl protein IDs of the CXCR4 gene. Among these SNPs, 200 SNPs were synonymous, 406 were missense or non-synonymous, 1,199 were in the intron region including 739 were in the 5’ (554) and 3’ (185) UTR regions. However, for the highly annotated protein with the Ensembl protein ID ENSP00000241393, only 94 non-synonymous SNPs were available. Subsequently, 2,322 SNPs for the CXCR4 gene were retrieved from the Ensembl database. Among these, 181 SNPs were identified as missense or non-synonymous variants, 208 as synonymous, 1,063 were intron variants, and 203 SNPs were located in the UTR (47 in the 5’ UTR and 156 in the 3’ UTR) regions. Combining data from these two distinct databases, a total of 204 non-synonymous SNPs were gathered for further analysis.

### Prediction of deleterious nsSNPs of CXCR4

**Fig 2:**
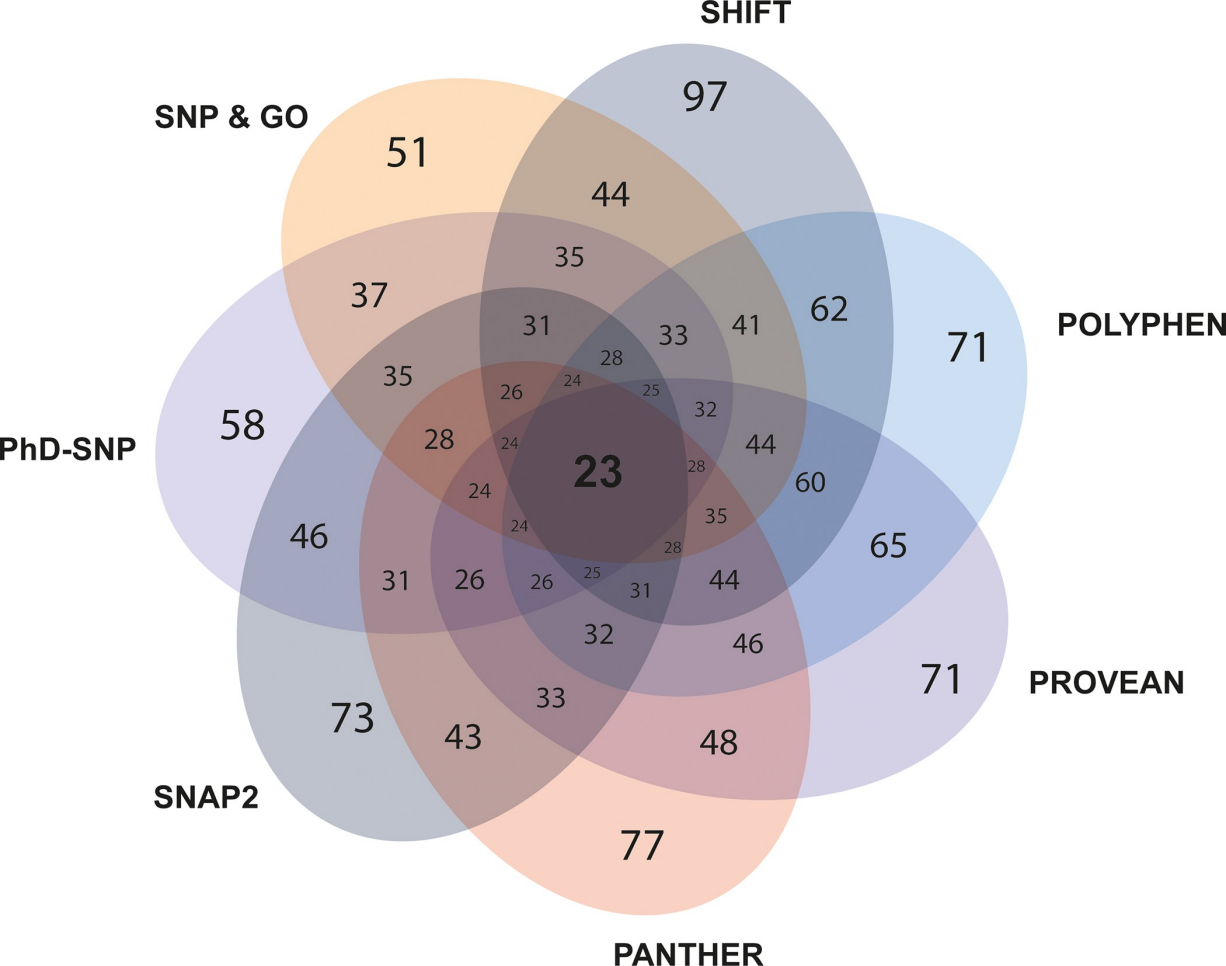
Most deleterious and disease-causing nsSNPs estimated by different in silico tools. Five in silico tools–SIFT, Polyphen2.0, PROVEAN, PANTHER, and SNAP2.0 were used to predict the most deleterious nsSNPs, and two tools–PhD-SNP and SNPs & GO–were used to predict disease-causing nsSNPs. Among the deleterious and disease-causing nsSNPs, 23 were common.

To assess the potentially deleterious effects of the 204 retrieved nsSNPs within the CXCR4 gene, 5 different tools–SIFT, Polyphen2.0, PROVEAN, PANTHER andSNAP2.0 were used (Fig 2). In the SIFT server, among the 204 nsSNPs, 97 variants were predicted as ‘damaging’, while 106 were tolerated. Moving to the PolyPhen2.0 web tool, 199 SNPs were found in the database. Further examination using the HumDiv section of Polyphen2.0 indicated that 71 variants as ’Probably damaging’, 31 variants as ’Possibly damaging’, and the remaining 97 variants as ’Benign’. Besides, in the HumVar section of the Polyphen 2.0, 70 amino acid variants were predicted as ’Probably damaging’, 32 as ’Possibly damaging’ and the remaining 97 as ‘Benign’. Both HumDiv and HumVar analysis revealed that 51.25% of the SNPs were predicted to have damaging effects, which were considered for further analysis. Out of 204 nsSNPs, 199 SNPs were found in the PROVEAN tool and these were then analyzed in this tool. In the PROVEAN web tool, only 71 variants were predicted as ‘Deleterious’, while the remaining 128 variants were predicted as ‘Neutral’. Consequently, 35.68% of the nsSNPs have a deleterious effect on CXCR4. Out of 204 nsSNPs, again 199 were available in PANTHER. and damaging effects of these variants were further analyzed in this tool. According to the preservation time, this tool predicted that 77 variants were classified as ’Probably Damaging’, 31 as ’Possibly Damaging’, and 91 as ’Probably Benign’, accounting for 54.27% of amino acid variants manifesting damaging effects on the CXCR4 protein. However, 204 SNPs were available in SNAP2.0 and among these 73 (35.78%) variants were predicted to have damaging effects on the protein. Combining the results from these 5 distinct tools yielded 30 nsSNPs with damaging effects on the CXCR4 protein (Fig 2).

### Prediction of disease-causing nsSNPs

SNPs are common genetic variations with implications for the susceptibility to many human diseases [57, 58]. To decipher whether nsSNPs within the CXCR4 gene have any ‘Disease causing’ effects we utilized web-based computational tools that include PhD-SNP, SNPs & GO. In the PhD-SNP, among the 204 nsSNPs, 58 (28.43%) variants were predicted as ‘Disease causing’ while the remaining were ‘Neutral’ (Fig 2). Moreover, SNP & GO provided prediction for all the 204 variants where only 51 (25%) were predicted as ‘Disease causing’ (Fig 2). When both analyses were integrated, we found 37 nsSNPs that were consistently predicted to be disease-causing (Fig 2). Among the characterized 30 deleterious and 37-disease causing nsSNPs of the CXCR4, 23 nsSNPs were found to be both deleterious and disease-causing, as commonly reported by all the seven different tools. (Fig 2 and Table 1). These common 23 nsSNPs (C28R, F36C, P42R, T43N, G55V, N56D, N56S, T73M, H79P, L80P, D84N, V96D, V96G, Y103D, G105W, H113P, W161C, D193Y, C218S, C251S, F292I, P299L, P299S) were chosen for further investigation.

**Table 1:** Most deleterious and disease-causing nsSNPs retrieved from NCBI dbSNP and Ensembl databases.

rsID	SNPs	SHIFT (Prediction/Score)	Polyphen2.0 (Prediction/Score)	PROVEAN (Prediction/Score)	PANTHER	SNAP2.0 (Prediction/Score)	PhD-SNP	SNPs & GO
**rs1684872844**	C28R	Deleterious 0	Probably Damaging 1	Deleterious -7.211	Probably Damaging	Effect 48	Disease	Disease
**rs1684872067**	F36C	Deleterious 0	Probably Damaging 1	Deleterious -4.364	Probably Damaging	Effect 51	Disease	Disease
**rs1268474896**	P42R	Deleterious 0.03	Probably Damaging 1	Deleterious -5.495	Probably Damaging	Effect 70	Disease	Disease
**rs778969048**	T43N	Deleterious 0.01	Probably Damaging 0.996	Deleterious -2.43	Probably Damaging	Effect 33	Disease	Disease
**rs1684869258**	G55V	Deleterious 0	Probably Damaging 1	Deleterious -6.982	Probably Damaging	Effect 89	Disease	Disease
**rs751756723**	N56D	Deleterious 0	Probably Damaging 1	Deleterious -3.879	Probably Damaging	Effect 89	Disease	Disease
**rs1393307051**	N56S	Deleterious 0	Probably Damaging 1	Deleterious -3.879	Probably Damaging	Effect 85	Disease	Disease
**rs943396331**	T73M	Deleterious 0	Probably Damaging 1	Deleterious -4.839	Probably Damaging	Effect 53	Disease	Disease
**rs1573614635**	H79P	Deleterious 0	Probably Damaging 1	Deleterious -6.472	Probably Damaging	Effect 85	Disease	Disease
**rs1684866510**	L80P	Deleterious 0	Probably Damaging 1	Deleterious -6.17	Probably Damaging	Effect 84	Disease	Disease
**rs368016542**	D84N	Deleterious 0	Probably Damaging 1	Deleterious -4.419	Probably Damaging	Effect 76	Disease	Disease
**rs1397762931**	V96D	Deleterious 0	Probably Damaging 0.99	Deleterious -4.76	Probably Damaging	Effect 55	Disease	Disease
**rs1397762931**	V96G	Deleterious 0.03	Probably Damaging 0.997	Deleterious -4.63	Probably Damaging	Effect 50	Disease	Disease
**rs765237875**	Y103D	Deleterious 0.01	Probably Damaging 0.995	Deleterious -7.211	Probably Damaging	Effect 53	Disease	Disease
**rs1313967515**	G105W	Deleterious 0	Probably Damaging 0.996	Deleterious 10.922	Probably Damaging	Effect 83	Disease	Disease
**rs1684862940**	H113P	Deleterious 0	Probably Damaging 0.999	Deleterious -6.812	Probably Damaging	Effect 66	Disease	Disease
**rs1331289711**	W161C	Deleterious 0	Probably Damaging 1	Deleterious -11.412	Probably Damaging	Effect 82	Disease	Disease
**rs367718547**	D193Y	Deleterious 0.01	Probably Damaging 0.932	Deleterious -7.016	Probably Damaging	Effect 58	Disease	Disease
**rs756207760**	C218S	Deleterious 0	Probably Damaging 1	Deleterious -5.359	Probably Damaging	Effect 69	Disease	Disease
**rs1488294654**	C251S	Deleterious 0	Probably Damaging 0.998	Deleterious -6.76	Probably Damaging	Effect 71	Disease	Disease
**rs566813397**	F292I	Deleterious 0	Probably Damaging 0.992	Deleterious -10.922	Probably Damaging	Effect 72	Disease	Disease
**rs1379060376**	P299L	Deleterious 0	Probably Damaging 0.999	Deleterious -4.839	Probably Damaging	Effect 75	Disease	Disease
**rs1558836146**	P299S	Deleterious 0.01	Probably Damaging 0.995	Deleterious -4.839	Probably Damaging	Effect 76	Disease	Disease

### Protein stability alteration prediction

We further assessed the effects of the filtered deleterious and disease-causing 23 nsSNPs on the stability of the CXCR4 using online tools I-mutant 3.0 and MUpro (Table 2). Both of the tools provide DDG values (free energy change), where DDG<0 (kcal/Mole) indicates decreased stability while DDG>0 (kcal/Mole) indicates increased stability. Out of the 23 nsSNPs, 18 (78.26%) SNPs were recognized to decrease in the stability of the protein by I-mutant3.0, and 21 (91.30%) SNPs were recognized to decrease in the stability of the protein by MUpro.

**Table 2:** Scores of protein stability alteration caused by the selected nsSNPs predicted by I-Mutant3.0 and MUpro.

rsID	SNPs	I-mutant3.0		MUpro	
		Prediction	DDG value (Kcal/mol)	Prediction	Value (SVM)
**rs1684872844**	C28R	Decrease	-0.52	Decrease	-0.82514082
**rs1684872067**	F36C	Decrease	-1.81	Decrease	-1.7857105
**rs1268474896**	P42R	Decrease	-1.11	Decrease	-0.85263794
**rs778969048**	T43N	Decrease	-1.26	Decrease	-0.40976981
**rs1684869258**	G55V	Decrease	-0.32	Increase	0.28941966
**rs751756723**	N56D	Increase	-0.03	Decrease	-0.9114518
**rs1393307051**	N56S	Increase	-0.24	Decrease	-0.78957197
**rs943396331**	T73M	Decrease	-0.39	Decrease	-0.66463562
**rs1573614635**	H79P	Increase	0.23	Decrease	-1.1456256
**rs1684866510**	L80P	Decrease	-1.78	Decrease	-1.5954337
**rs368016542**	D84N	Decrease	-0.93	Decrease	-1.5365342
**rs1397762931**	V96D	Decrease	-1.56	Decrease	-1.6774303
**rs1397762931**	V96G	Decrease	-2.33	Decrease	-2.7313111
**rs765237875**	Y103D	Decrease	-1.56	Decrease	-1.6774303
**rs1313967515**	G105W	Decrease	-0.88	Decrease	-0.77741612
**rs1684862940**	H113P	Increase	0	Decrease	-0.74277823
**rs1331289711**	W161C	Decrease	-1.74	Increase	0.1777163
**rs367718547**	D193Y	Decrease	-0.2	Decrease	-1.0231434
**rs756207760**	C218S	Increase	0.27	Decrease	-1.6493399
**rs1488294654**	C251S	Decrease	-1.12	Decrease	-1.7105409
**rs566813397**	F292I	Decrease	-0.47	Decrease	-1.1101731
**rs1379060376**	P299L	Decrease	-1.33	Decrease	-0.11751985
**rs1558836146**	P299S	Decrease	-1.08	Decrease	-1.2798982

### Conservation and surface accessibility of nsSNPs of CXCR4

**Fig 3:**
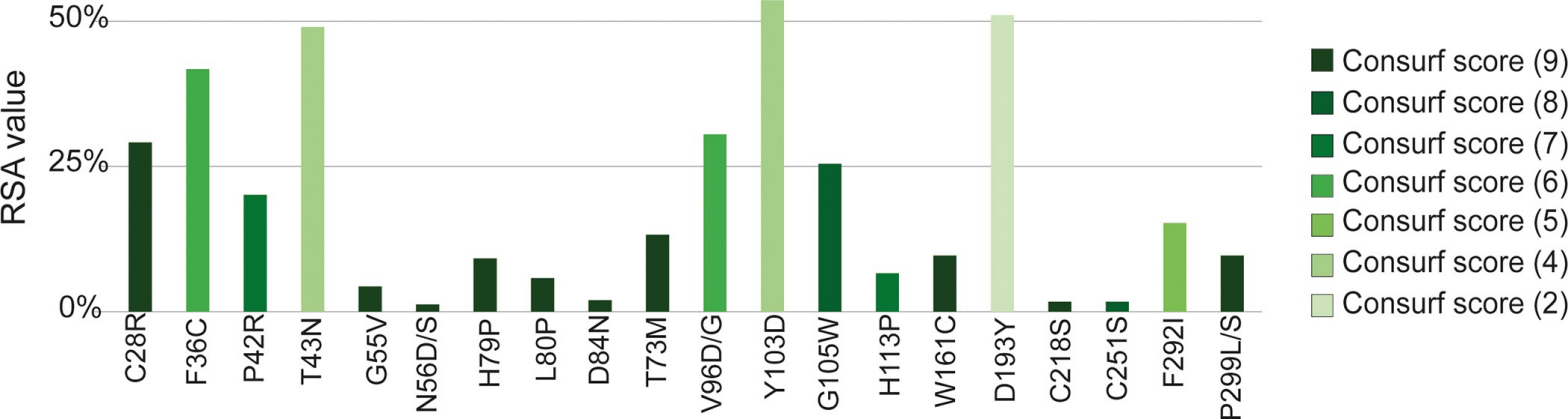
Schematic representation of the relative surface accessibility and conservation of the high-risk SNPs predicted by NetSurfP-2.0 and Consurf web tool. In NetSurfP2.0, a threshold of 25% was utilized, meaning SNPs with more than 25% RSA are expected to be exposed on the protein surface.

Preservation of conserved regions is essential for the structural integrity and function of specific proteins as any alteration within these regions can cause more significant and deleterious effects compared to alterations in the non-conserved regions. To locate whether the 23 filtered nsSNPs are in conserved or non-conserved regions of the CXCR4 protein, we used the Consurf web tool (Fig 3 and Table 3). Nineteen nsSNPs of the filtered 23 nsSNPs were predicted to be located within the conserved region of the protein. Notably, these 16 nsSNPs exhibited varying conservation scores, with 12 nsSNPs possessing a score of 9 (denoting height degree of conservation), while 2 had a score of 8, 2 with a score of 7, 3 with a score of 6, 1 with a score of 4, and 1 with a score of 2 (Fig 3 and Table 3). Surface accessibility is an essential criterion for receptor proteins like CXCR4. We used NetSurfP-2.0 to analyze the surface accessibility of the mutated amino acids of CXCR4 (Table 3). Among the filtered most deleterious and disease-causing amino acids, 8 amino acids were predicted to be exposed to the surface of the protein, while the remaining 15 were predicted to be buried within its structure.

**Table 3:** The conservation and surface accessibility of selected nsSNPs by Consurf & Netsurf 2.0.

rsID	SNPs	Consurf		Netsurf2.0	
		Score of conservation	Conservation	Probability of disorder	Buried/Exposed
**rs1684872844**	C28R	9	Conserved	0.101566032	exposed
**rs1684872067**	F36C	6	Conserved	0.035028115	exposed
**rs1268474896**	P42R	7	Conserved	0.000158129	buried
**rs778969048**	T43N	4	Variable	0.000269237	exposed
**rs1684869258**	G55V	9	Conserved	0.000145211	buried
**rs751756723**	N56D	9	Conserved	3.31E-05	buried
**rs1393307051**	N56S	9	Conserved	3.31E-05	buried
**rs943396331**	T73M	9	Conserved	0.00023658	buried
**rs1573614635**	H79P	9	Conserved	3.03E-05	buried
**rs1684866510**	L80P	9	Conserved	3.15E-05	buried
**rs368016542**	D84N	9	Conserved	2.25E-05	buried
**rs1397762931**	V96D	6	Conserved	0.000632308	exposed
**rs1397762931**	V96G	6	Conserved	0.000632308	exposed
**rs765237875**	Y103D	4	Average	0.006736517	exposed
**rs1313967515**	G105W	8	Conserved	0.003654183	exposed
**rs1684862940**	H113P	7	Conserved	3.25E-05	buried
**rs1331289711**	W161C	9	Conserved	2.52E-05	buried
**rs367718547**	D193Y	2	Variable	0.003941391	exposed
**rs756207760**	C218S	9	Conserved	0.000144533	buried
**rs1488294654**	C251S	8	Conserved	0.000216715	buried
**rs566813397**	F292I	5	Average	0.000363415	buried
**rs1379060376**	P299L	9	Conserved	3.18E-05	buried
**rs1558836146**	P299S	9	Conserved	3.18E-05	buried

### Post-translational modification caused by nsSNPs of CXCR4

**Fig 4:**
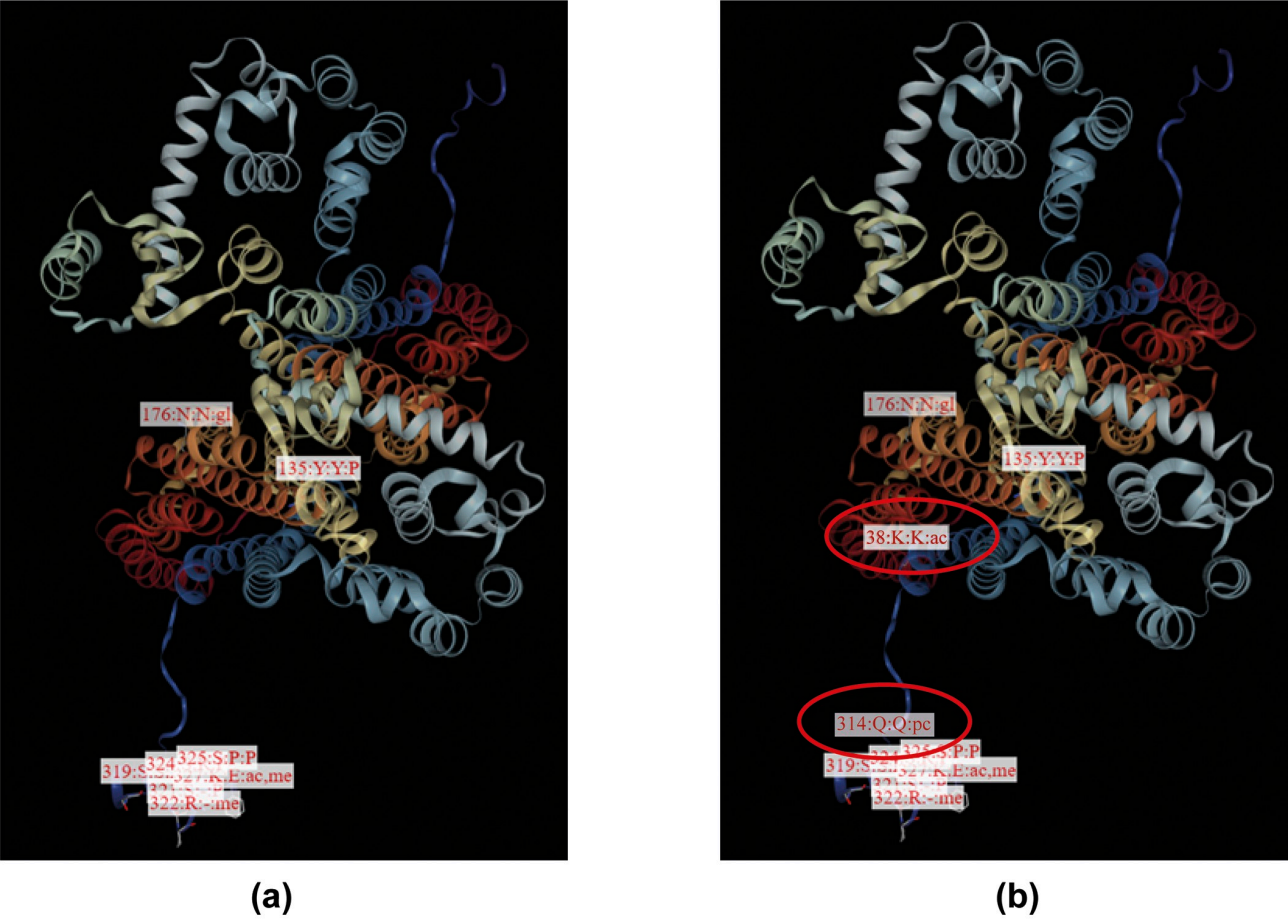
Effects of the filtered nsSNPs within CXCR4 on post-translational modification. (a) Within the wild-type of CXCR4 protein, K38, and Q314 do not exhibit acetylation or Pyrrolidone carboxylic acid modification, while (b) in the context of mutated protein with F36C and P299L substitution, K38 becomes acetylated and Q314 undergoes pyrrolidone carboxylic acid modification.

Post-translational modifications (PTM) can change the protein structure and thus can destabilize proteins. To predict the effects of the most deleterious and disease-causing nsSNPs on PTM, we employed the MusiteDeep server (Fig 4). For the CXCR4 protein, the tool predicted 26 amino acid residues susceptible to 5 different types of PTMs (confidence score 0.5)–Glycosylation, Phosphorylation, Acetylation, Methylation, and Ubiquitination. In the mutated condition of the protein, in addition to these 26 amino acid residues susceptible to PTM, the tool predicted the modification at 2 extra amino acid residues–K38 and Q314- indicating these two modifications as acetylation and Pyrrolidone carboxylic acid, respectively, while this modification was found to be absent in the wild-type of the protein. Notably, the P36C substitution may impact the acetylation of the K38 reside, potentially due to the proximity of cysteine to lysine, a factor recognized to enhance lysine N-acetylation [59]. Furthermore, due to the distinct cyclic structure of proline, it is known to confer structural rigidity [60]. Its proximity to glutamine may have an impact on the accessibility and local structure of the protein. Thus, the P299 residue in the wild protein may influence the activity of the glutaminyl cyclase (QC) enzyme, which converts Pyrrolidone carboxylic acid (PCA) to pyroglutamic acid (pGlu) residues. However, in the mutated protein, the substitution of P299 to L299 appears to facilitate this enzymatic conversion.

### Effects of the nsSNPs on the phenotype of CXCR4

To decipher whether the filtered 23 nsSNPs within the CXCR4 protein have any phenotypic effect, we employed 2 distinct databases–MutPred2 and Project HOPE. MutPred2 was employed to predict a spectrum of molecular mechanisms that could ensure from these nsSNPs in mutated proteins, encompassing alteration in metal binding, loss or gain of catalytic or allosteric sites, loss or gain of GPI-anchor amidation, loss or gain of helix, loop or strand, alteration in the interface, and any other kind of alteration (predicted with probability values or p values) (Table 4). Remarkably, all but two variants, T43N and D193Y in CXCR4protein, were predicted to be pathogenic as they scored >0.5 in MutPred2 predictions. The Project-HOPE, on the other hand, predicted the effects of these nsSNPs on the size, hydrophobicity, charge, and bond formation of the protein. It showed that out of 23 nsSNPs examined, 8 resulted in the substitution of larger amino acids, while 8 led to the substitution of smaller amino acids. Moreover, 11 amino acid substitutions decreased the hydrophobicity, while 3 increased it, additionally, 2 changes shifted the charge from neutral to positive, 3 from neutral to negative, and 1 from negative to neutral (Table 5). These residues are strategically located in a domain that is important for the activity of the protein and are in contact with residues within another domain. Consequently, for the residues buried in the core of the protein, substitutions with larger amino acid residues may disrupt their compatibility and thereby compromise the structural integrity of the protein. Similarly, for the residues located on the protein surface, mutation with bigger amino acid residues may impede interactions with other molecules. Conversion from neutral to positively charged amino acid could provoke electrostatic repulsion, while conversions from neutral to negatively charged amino acids might disrupt chemokine binding and protein folding. Alterations in hydrophobicity may further exacerbate potential complications in interaction with other molecular entities.

**Table 4:** Effects of the selected nsSNPs on the structure and function of the protein predicted by Mutpred2.0.

rsID	SNPs	Mutpred			
		Score	Effects	Probability	P-value
**rs1684872844**	C28R	0.897	Loss of Disulfide linkage at C28	1.00	8.3e-05
			Gain of Intrinsic disorder	0.44	4.7e-03
			Altered Disordered interface	0.39	5.9e-03
			Altered Transmembrane protein	0.29	2.7e-04
			Altered Metal binding	0.28	0.02
			Gain of SUMOylation at K25	0.19	0.04
**rs1684872067**	F36C	0.867	Altered Disordered interface	0.34	0.01
			Altered Transmembrane protein	0.31	1.1e-04
**rs1268474896**	P42R	0.859	Altered Transmembrane protein	0.36	2.4e-05
			Gain of Helix	0.28	0.02
			Altered Disordered interface	0.27	0.04
			Altered Ordered interface	0.26	0.02
**rs778969048**	T43N	0.426	-	-	
**rs1684869258**	G55V	0.89	Loss of Catalytic site at N56	0.09	0.04
			Altered Transmembrane protein	0.01	9.0e-03
			Loss of GPI-anchor amidation at N56	0.01	0.03
**rs751756723**	N56D	0.826	Loss of Catalytic site at N56	0.09	0.04
			Altered Transmembrane protein	0.01	0.01
			Loss of GPI-anchor amidation at N56	0.01	0.03
**rs1393307051**	N56S	0.717	Loss of Catalytic site at N56	0.09	0.04
			Altered Transmembrane protein	0.02	7.3e-03
			Loss of GPI-anchor amidation at N56	0.01	0.03
**rs943396331**	T73M	0.503	Altered Ordered interface	0.31	2.1e-03
			Altered Transmembrane protein	0.27	5.4e-04
			Gain of Helix	0.27	0.05
**rs1573614635**	H79P	0.932	Altered Ordered interface	0.34	5.3e-03
			Altered Transmembrane protein	0.30	1.8e-04
			Gain of Allosteric site at Y76	0.21	0.03
			Altered Metal binding	0.15	0.04
**rs1684866510**	L80P	0.954	Altered Ordered interface	0.31	2.0e-03
			Altered Transmembrane protein	0.30	1.4e-04
			Loss of Helix	0.29	0.01
			Gain of Strand	0.29	0.04
			Altered Metal binding	0.14	0.04
**rs368016542**	D84N	0.877	Altered Transmembrane protein	0.28	3.8e-04
			Altered Metal binding	0.13	0.05
**rs1397762931**	V96D	0.918	Altered Transmembrane protein	0.48	0.0e+00
			Gain of Relative solvent accessibility	0.35	1.4e-03
			Gain of Loop	0.26	0.05
			Altered Ordered interface	0.24	0.04
**rs1397762931**	V96G	0.863	Altered Transmembrane protein	0.33	6.3e-05
			Gain of Relative solvent accessibility	0.27	0.02
			Altered Ordered interface	0.24	0.04
			Altered Stability	0.13	0.03
**rs765237875**	Y103D	0.858	Altered Ordered interface	0.53	1.0e-04
			Gain of Relative solvent accessibility	0.33	2.5e-03
			Gain of Allosteric site at W102	0.20	0.04
			Altered Metal binding	0.18	0.05
**rs1313967515**	G105W	0.88	Loss of Relative solvent accessibility	0.32	4.7e-03
			Altered Ordered interface	0.28	5.4e-03
			Altered Metal binding	0.27	0.02
			Loss of Strand	0.26	0.04
			Altered Transmembrane protein	0.25	1.3e-03
			Loss of Disulfide linkage at C109	0.20	0.02
**rs1684862940**	H113P	0.882	Altered Metal binding	0.27	0.02
			Loss of Helix	0.27	0.04
			Gain of Strand	0.27	0.03
			Altered Transmembrane protein	0.25	1.5e-03
			Altered Ordered interface	0.24	0.03
			Gain of Disulfide linkage at C109	0.20	0.02
**rs1331289711**	W161C	0.946	Altered Ordered interface	0.27	7.6e-03
**rs367718547**	D193Y	0.426	-	-	-
**rs756207760**	C218S	0.92	Gain of Allosteric site at Y219	0.26	7.6e-03
			Altered Ordered interface	0.24	0.04
			Altered Transmembrane protein	0.01	0.01
**rs1488294654**	C251S	0.874	Altered Transmembrane protein	0.27	8.6e-04
			Altered Ordered interface	0.24	0.04
**rs566813397**	F292I	0.791	Altered Transmembrane protein	0.13	0.02
**rs1379060376**	P299L	0.902	Altered Ordered interface	0.25	0.02
			Altered Transmembrane protein	0.13	0.02
**rs1558836146**	P299S	0.872	Altered Ordered interface	0.25	0.03
			Altered Transmembrane protein	0.13	0.02

**Table 5:** Effects of the nsSNPs in the amino acid changes of the mutated protein as predicted by the Project-HOPE.

rsID	SNPs	Difference in size	Hydrophobicity	Charge Change
**rs1684872844**	C28R	Bigger	Decreased	Neutral to Positive
**rs1684872067**	F36C	Smaller	N/A	N/A
**rs1268474896**	P42R	Bigger	Decreased	Neutral to Positive
**rs778969048**	T43N	Bigger	Decreased	N/A
**rs1684869258**	G55V	Bigger	Increased	N/A
**rs751756723**	N56D	N/A	N/A	Negative to Neutral
**rs1393307051**	N56S	Smaller	Increased	N/A
**rs943396331**	T73M	Bigger	Decreased	N/A
**rs1573614635**	H79P	Smaller	Increased	N/A
**rs1684866510**	L80P	Smaller	N/A	N/A
**rs368016542**	D84N	N/A	N/A	Negative to Neutral
**rs1397762931**	V96D	Bigger	Decreased	Neutral to Negative
**rs1397762931**	V96G	Smaller	Increased	N/A
**rs765237875**	Y103D	Smaller	Decreased	Neutral to Negative
**rs1313967515**	G105W	Bigger	N/A	N/A
**rs1684862940**	H113P	Smaller	Increased	N/A
**rs1331289711**	W161C	Smaller	N/A	N/A
**rs367718547**	D193Y	Bigger	Increased	Negative to Neutral
**rs756207760**	C218S	N/A	Decreased	N/A
**rs1488294654**	C251S	N/A	Decreased	N/A
**rs566813397**	F292I	Smaller	Decreased	N/A
**rs1379060376**	P299L	Bigger	N/A	N/A
**rs1558836146**	P299S	Smaller	Decreased	N/A

### Structural alteration and difference between the normal and mutated proteins

In the investigation of the secondary structure of both the normal and mutated variants of CXCR4, the SOMPA tool was employed. It was found that within the 352 amino acids constituting the protein, 184 amino acids (52.27%) were positioned within α helical region, 8 amino acids (2.27%) within β turn structures, 105 amino acids (29.83%) within random coil configuration and 55 amino acids (15.62%) within the extended strand. Notably, specific mutations were observed with these structural elements. The F36C, P42R, T43N, T73M, H79P, L80P, D84N, V96D, V96G, Y103D, G105W, H113P, W161C, D193Y, C218S, F292I, P299L, and P299S mutations were situated within α helical regions, whereas G55V, N56D, N56S, and C251S mutations were within β turn structures, and C28R and P42R mutations were found within random coil configurations.

Subsequently, the most deleterious nsSNPs of the CXCR4 protein were analyzed in the Mutation3D server for predicting mutation clusters of the nsSNPs. The server showed that all the mutations were clustered in 7 distinct sections (Table 6), Remarkably, it was also noted that the protein encoded by CXCR4 is encompassed within the set of known cancer-associated genes.

**Table 6:** Clusters of the mutation predicted by Mutation3D webserver.

Cluster no.	P-Value	SNPs
**1**	2.94e-02	G55V, N56D, N56S, P299L, P299S
**2**	1.21e-01	G55V, N56D, N56S
**3**	3.64e-01	P42R, T43N, V96D, V96G
**4**	1.09e+00	H79P, L80P, D84N, W161C
**5**	1.09e+00	V96D, V96G, Y103D, G105W
**6**	1.18e+00	C251S, F292I, P299L, P299S
**7**	1.27e+00	F36C, P42R, T43N

To assess the impact of the selected nsSNPs on the structural conformation of the CXCR4 protein, we conducted homology modeling using a web server–SWISS-MODEL. At first, we employed this server to predict the structure of the wild-type protein. Being a homology modeling server, it relies on the designated PDB protein as its template. From the numerous predicted models provided by the server, we selected the model with the highest coverage and sequence identity, accounting for 0.82 and 87.29%, respectively. For this selection, the template structure 3odu.1.A was employed. This model had a QMEAN Z-Score of -5.18, a MolProbity Score of 1.36, and 97.48% Ramachandran-Favoured regions with no Ramachandran Outliers. For each mutated protein structure, we individually substituted every mutant amino acid within the wild-type CXCR4 sequence. Subsequently, both the wild-type and mutated protein sequences were separately submitted to SWISS-MODEL for homology modeling using the previous way (Table 7). Following the modeling process, we rigorously validated the structures for the wild-type and mutant variants of the CXCR4 using various tools, including Swiss model assessment, ERRAT, and PROCHECK (Table 8).

**Table 7:** Different values of the homology modeling of wild type and mutated protein designed through SWISS MODEL webserver.

rsIDs	SNPs	Seq Identity	Seq Similarity	Coverage	QMEAN Z-Scores	MolProbity Score
**N/A**	N/A	99.04%	0.61	0.89	-4.03	1.85
**rs1684872844**	C28R	98.72%	0.61	0.89	-3.94	1.88
**rs1684872067**	F36C	98.72%	0.61	0.89	-3.93	1.88
**rs1268474896**	P42R	98.72%	0.61	0.89	-3.88	1.83
**rs778969048**	T43N	98.72%	0.61	0.89	-3.92	2.03
**rs1684869258**	G55V	98.72%	0.61	0.89	-4.09	1.87
**rs751756723**	N56D	98.72%	0.61	0.89	-4.02	1.87
**rs1393307051**	N56S	98.72%	0.61	0.88	T4-3.87	1.85
**rs943396331**	T73M	98.71%	0.61	0.89	-4.11	1.9
**rs1573614635**	H79P	98.72%	0.61	0.89	-4.02	1.91
**rs1684866510**	L80P	98.72%	0.61	0.89	-3.94	1.85
**rs368016542**	D84N	98.72%	0.61	0.89	-3.94	1.81
**rs1397762931**	V96D	98.72%	0.61	0.89	-4.01	1.81
**rs1397762931**	V96G	98.72%	0.61	0.89	-3.91	1.81
**rs765237875**	Y103D	98.72%	0.61	0.89	-4.23	1.94
**rs1313967515**	G105W	98.71%	0.61	0.89	-4.04	1.84
**rs1684862940**	H113P	98.72%	0.61	0.89	-3.97	1.84
**rs1331289711**	W161C	98.72%	0.61	0.89	-4.06	1.84
**rs367718547**	D193Y	98.71%	0.61	0.89	-3.95	1.85
**rs756207760**	C218S	98.72%	0.61	0.89	-3.92	1.87
**rs1488294654**	C251S	98.72%	0.61	0.89	-3.96	1.89
**rs566813397**	F292I	98.71%	0.61	0.89	-3.88	1.85
**rs1379060376**	P299L	98.71%	0.61	0.89	-3.86	1.87
**rs1558836146**	P299S	98.71%	0.61	0.89	-4.03	1.85

**Table 8:** Structure validation of the formed protein structure provided by the SWISS-MODEL web server.

rsIDs	SNPs	Ramachandran Favoured	ERRAT	PROCHECK
**N/A**	N/A	96.72%	95.1941	Errors: 3, Warning: 2, Pass: 3
**rs1684872844**	C28R	96.72%	95.203	Errors: 3, Warning: 2, Pass: 3
**rs1684872067**	F36C	96.55%	95.5638	Errors: 3, Warning: 2, Pass: 3
**rs1268474896**	P42R	96.55%	95.9484	Errors: 3, Warning: 2, Pass: 3
**rs778969048**	T43N	96.72%	95.1941	Errors: 1, Warning: 5, Pass: 2
**rs1684869258**	G55V	96.55%	95.5801	Errors: 3, Warning: 2, Pass: 3
**rs751756723**	N56D	96.55%	95.1852	Errors: 3, Warning: 2, Pass: 3
**rs1393307051**	N56S	96.55%	95.5638	Errors: 3, Warning: 2, Pass: 3
**rs943396331**	T73M	96.72%	95.1941	Errors: 3, Warning: 2, Pass: 3
**rs1573614635**	H79P	96.55%	96.1326	Errors: 3, Warning: 3, Pass: 2
**rs1684866510**	L80P	96.55%	96.1255	Errors: 3, Warning: 2, Pass: 3
**rs368016542**	D84N	96.72%	95.5638	Errors: 3, Warning: 2, Pass: 3
**rs1397762931**	V96D	96.72%	95.1941	Errors: 3, Warning: 2, Pass: 3
**rs1397762931**	V96G	96.72%	95.1941	Errors: 3, Warning: 2, Pass: 3
**rs765237875**	Y103D	96.72%	95.1941	Errors: 3, Warning: 2, Pass: 3
**rs1313967515**	G105W	96.38%	95.5556	Errors: 4, Warning: 2, Pass: 2
**rs1684862940**	H113P	96.72%	94.9907	Errors: 3, Warning: 2, Pass: 3
**rs1331289711**	W161C	96.72%	95.5638	Errors: 3, Warning: 2, Pass: 3
**rs367718547**	D193Y	96.72%	95.1941	Errors: 3, Warning: 2, Pass: 3
**rs756207760**	C218S	96.72%	95.1941	Errors: 3, Warning: 2, Pass: 3
**rs1488294654**	C251S	96.55%	95.3704	Errors: 3, Warning: 2, Pass: 3
**rs566813397**	F292I	96.55%	95.7486	Errors: 1, Warning: 4, Pass: 3
**rs1379060376**	P299L	96.90%	95.3789	Errors: 3, Warning: 2, Pass: 3
**rs1558836146**	P299S	96.55%	95.1941	Errors: 3, Warning: 2, Pass: 3

**Fig 5:**
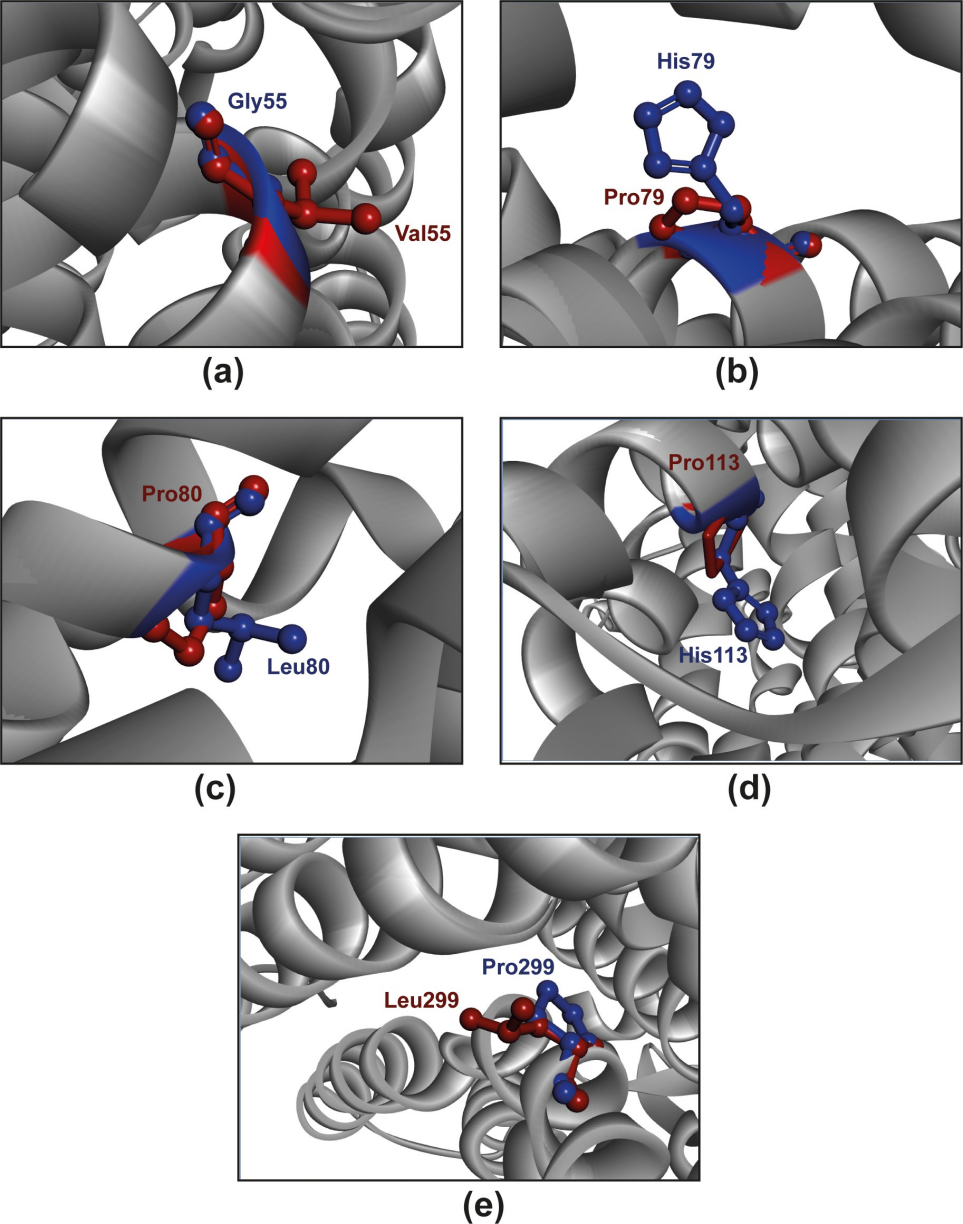
Superimposed structures of wild and mutant variants of CXCR4. (a) G55V, (b) H79P, (c) L80P, (d) H113P, and (e) P299L. Here, the blue-colored residues represent the native amino acids, and the red residues represent mutated residues. The superimposed position of the native and mutated amino acids shows that the distinguished structure of the side chain of these amino acids likely have impact on the alteration of the 3D structure of the protein.

After validation, the structures of mutated proteins were analyzed in TM-align, PyMoL, and BIOVIA discovery studio visualizer to evaluate the TM and RMSD scores. Notably, among these structures, the G55V, H79P, L80P, H113P, and P299L mutations exhibited minimum TM scores, measuring 0.99992, 0.99998, 0.99996, 0.99998, and 0.99995, respectively. Conversely, these mutations displayed the maximum RMSD values with PyMoL measuring values of 0.007, 0.013, 0.008, 0.006 and 0.004, while the BIOVIA discovery studio visualizer recorded values of 0.053, 0.038, 0.046, 0.043 and 0.051 (Fig 5). It is essential to note that a lower TM score indicates less topological similarity while a higher RMSD indicates a larger disparity between the wild-type and mutant structures. Based on the previous prediction about the anticipated effects of the nsSNPs on phenotype, structure, and clustering, we considered G55V, H79P, L80P, H113P, and P299L for further in-depth visualization.

**Fig 6:**
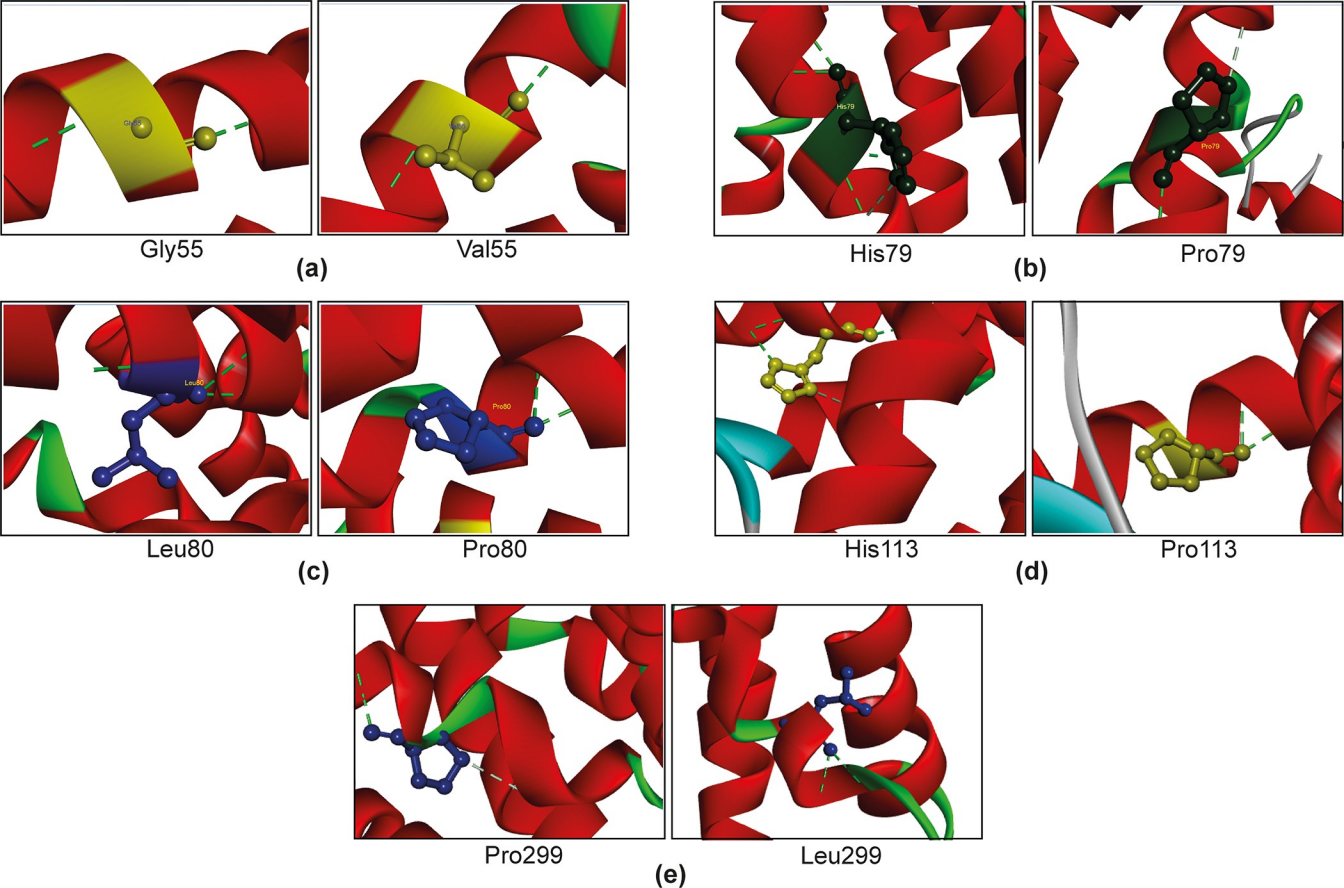
Structural analysis of wild type and mutated variants of CXCR4. (a) G55 in the wild-type protein and Val55 in mutated protein forms 2 H-bonds with Thr51 and Val59; (b) His79 in wild type protein engages in 4 H-bonds with Lys75, Tyr76, Val82, and Ala83, while the mutated Pro79 forms only 1 H-bond with Ala83; (c) H-bond number again changed with Leu80 (with Tyr76, Ala83, and Asn84) mutation from 3 to 2 with Pro80 (with Ala83 and Ans84); meanwhile, (d) H-bond number decreased from 4 to 2 in the mutated version of H113 (Cys109, Cys109, Thr117, Asp171) to P113 (Tyr116, Thr117); (e) P299 and L299 both create 2 H-bond but with different amino acids, P299 with Tyr302, Cys295, and L299 with Tyr302, Ala303, respectively. The changes in H-bond in the 3D structure of the CXCR4 protein likely have a significant impact on the structure functions of the protein.

Besides RMSD values, the superimposed structures were further analyzed to evaluate the differences in the number of H-bonds (Fig 6). Furthermore, hydrophobicity in the BIOVIA discovery studio visualizer (Table 9).

**Table 9:** Chemical analysis of the selected nsSNPs by BIOVIA discovery studio visualizer.

rsIDs	Amino acid position	Residues		Hydrophobicity	Hydrogen bond number
**rs1684869258**	55	Wild	Glycine (G)	-0.4	2 (55G-51T, 59V-55G)
		Mutant	Valine (V)	4.2	2 (55V-51T, 59V-55V)
**rs1573614635**	79	Wild	Histidine (H)	-3.2	4 (79H-75K, 79H-76Y, 82V-79H,83A-79H)
		Mutant	Proline (P)	-1.6	1 (83A-79P)
**rs1684866510**	80	Wild	Leucine (L)	3.8	3 (80L-76Y, 83A-80L, 84N-80L)
		Mutant	Proline (P)	-1.6	2 (83A-80P, 84N-80P)
**rs1684862940**	113	Wild	Histidine (H)	-3.2	4 (113H-109C, 113H-109C, 117T-113H, 113H-171D)
		Mutant	Proline (P)	-1.6	2 (116Y-113P, 117T-113H)
**rs1379060376**	299	Wild	Proline (P)	-1.6	2 (302Y-299P, 299P-295C)
		Mutant	Leucine (L)	3.8	2 (302Y-299L, 303A-299L)

### Effects of non-coding SNPs of CXCR4

SNPs located within the non-coding regions of a gene can influence gene expression by affecting regulatory elements. In this study, 1,199 SNPs were identified in the non-coding regions of the CXCR4 gene using the dbSNP database, while the Ensembl database reported 1,266 SNPs in these regions. The potential functional impact of these non-coding SNPs was assessed using RegulomeDB, which provided a detailed analysis of their regulatory roles. Out of the 1,266 SNPs in non-coding regions, 584 were categorized into eight functional ranks: 1b, 1f, 2a, 2b, 2c, 3a, 4, and 5. The majority of these SNPs were predicted to influence transcription factor binding sites and chromatin accessibility. A detailed breakdown of the number and percentage of SNPs in each rank is presented in S1 Table and S1 Fig, respectively, while information about the specific criteria for each rank is summarized in S2 Table.

To further know the potential impact of SNPs on the binding of CXCR4 targeted miRNA, our focus was narrowed to ’SNPs located exclusively within the 3’ UTR as this is the usual site for miRNA targeting. We conducted the assessment by retrieving SNPs located within the 3’ UTR region using the PolymiRTS database. Among the initial 126 SNPs identified within this region, the server provided information for only 5 SNPs—rs112957293, rs148300422, rs17848059, rs17848060, and rs1804029. These 5 variants belonged to ‘’D’’ and ‘’C’’ functional classes, indicating that these derived alleles might disrupt conserved miRNA binding sites or create new ones. All those 5 variants were also found in RegulomeDB. In RegulomeDB, these 5 SNPs were assigned rank numbers, with values 5, 4, 6, 4, and 7 and corresponding scores of 1.0, 0.61, 0.0, 0.61 and 0.18, respectively. As rs112957293 (1.0), rs148300422 (0.61), and rs17848060 (0.61) scores near to 1, these SNPs could potentially hold significant implications for transcription factor binding within the ’3’ UTR region of the CXCR4 gene.

### Docking

**Fig 7:**
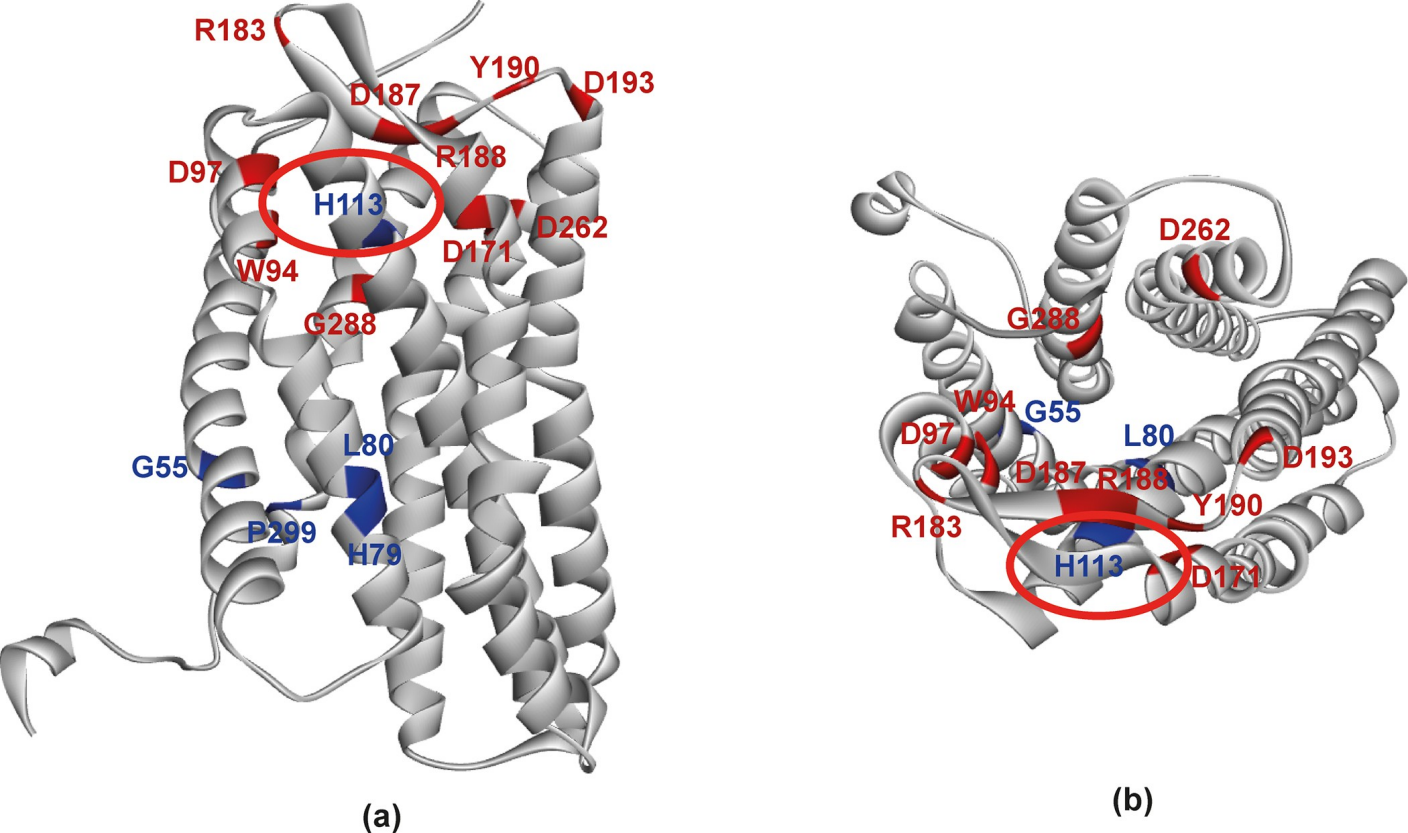
The structure of the protein shows the docking-related and mutation-prone residues. Docking-related residues (denoted with red) and the mutation-prone residues (denoted with blue) from both (a) side view and (b) top view. In both images, the residue H113, positioned in the docking site and susceptible to mutation is indicated by a red circle.

**Fig 8:**
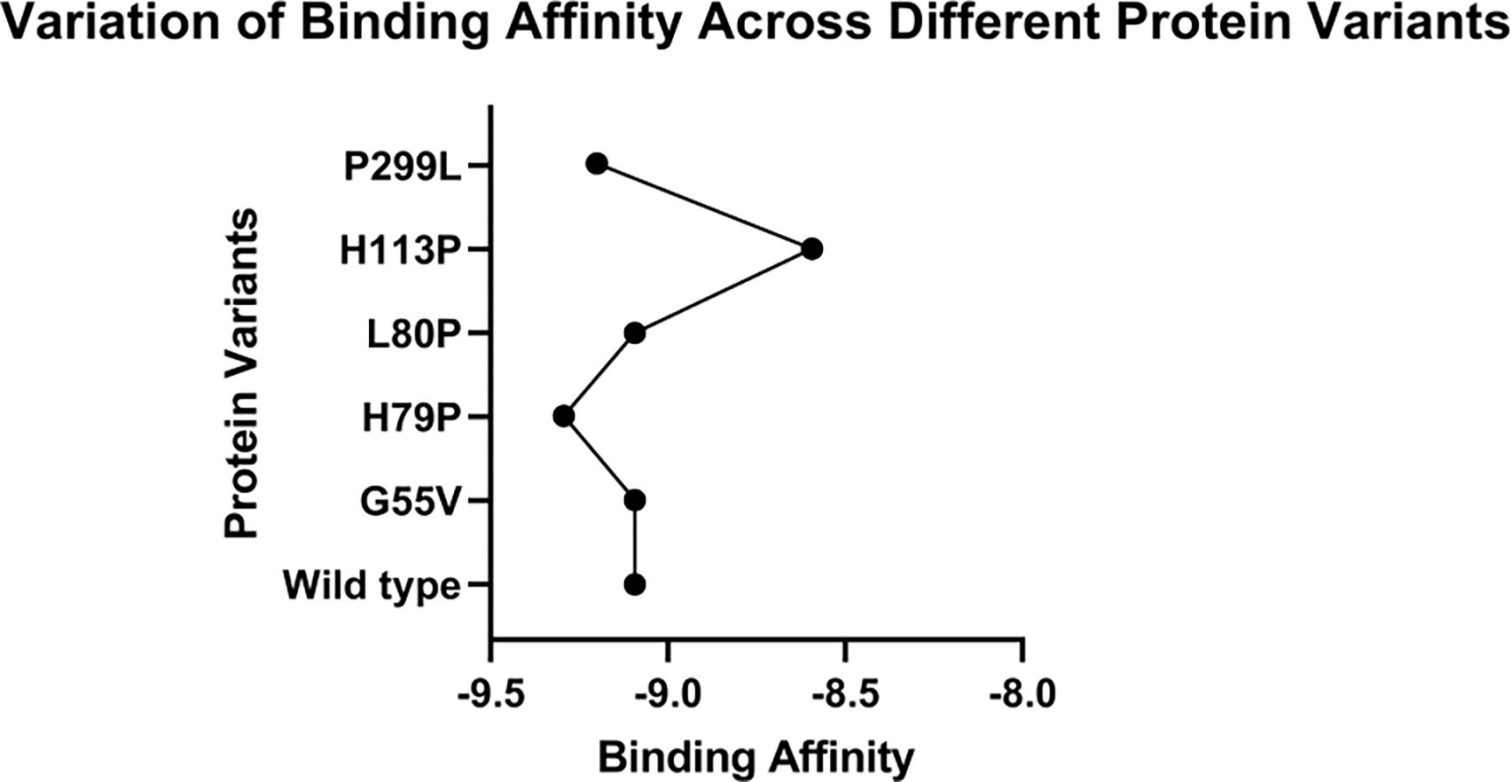
Molecular docking results of CXCR4-modulator 1 with CXCR4 protein using PyRx software. All the mutant variants of the ligand showed greater affinity with their receptor during the simulated docking process. However, a significant decrease in the affinity was observed for the H113P mutant variant.

CXCR4-modulator-1 (compound ZINC72372983) is known as a common ligand for antagonizing the activity of CXCR4 [61]. When the CXCR4 receptor binds with small molecules, key residues involved in this interaction include Trp94, Asp 97, Asp171, Arg183, Asp187, Arg188, Tyr190, Asp 193, Asp262, and Glu288 [62]. Thus, in our investigation, we conducted molecular docking by targeting the region of the molecule containing these critical residues. The docking was performed in the PyRx software, wherein a grid box with dimensions of 35×35×35 Å was centered at X: 15, Y: -7.5, and Z: 78 for each docking simulation. Consequently, this ligand was docked against both the wild type and mutated variants of CXCR4, the latter bearing deleterious nsSNPs. Our docking analysis revealed that only one SNP, specifically H113P, appeared to potentially affect the binding affinity due to its strategic location within the docking site (Fig 7). To investigate more closely into the implications of these mutations, five of the most prominently found mutants (G55V, H79P, L80P, H113P, and P299L), and the wild type CXCR4 were docked against this CXCR4-modulator 1. In PyRx, the results were expressed in terms of binding affinity. Notably, the G55V and H79P mutations exhibited similar binding affinity as that of wild-type molecules, while the L80P and P299L mutations showed slightly higher affinity. Conversely, the H113P mutation showed significantly less binding affinity than the wild type (Fig 8). Therefore, this suggests that the SNPs, mainly the H113P mutation, may exert significant effects on the function of the CXCR4 protein.

### Effect of non-harmful nsSNPs of CXCR4

As discussed, in the analysis of 181 nsSNPs of the CXCR4 gene, most were identified as deleterious or disease-causing. Using seven bioinformatics tools, 23 harmful nsSNPs were filtered. To assess sensitivity and specificity of the tools that were used for identification of deleterious or disease-causing nsSNPs, we also assessed the effects of non-harmful nsSNPs (those identified as non-deleterious and non-disease-causing) on the structure and function of CXCR4. Four nsSNPs—M24V, R30L, N35T, and I44V—were identified as non-harmful by these tools. Of these, only I44V was buried, while the others were surface-exposed, as predicted by Consurf 2.0 and Netsurf. Mutsitedeep indicated that none of these amino acids gained post-translational modifications after mutation. However, all mutated amino acids were smaller than their wild-type counterparts, potentially reducing external interactions, as projected by Project HOPE.

In addition, protein modeling was performed using SWISS-MODEL with 3odu.1.A as the template (coverage: 0.82, sequence identity: 87.29%). The model exhibited a QMEAN Z-Score of -5.18, a MolProbity Score of 1.36, and 97.48% of residues in favored regions with no outliers in the Ramachandran plot. The properties of the mutated proteins are detailed in Table 10. These models were also validated using Swiss model assessment, ERRAT, and PROCHECK (Table 11).

**Table 10:** Homology modeling values for wild-type and non-harmful nsSNP-mutated proteins designed using the SWISS-MODEL web server.

rsIDs	SNPs	Seq Identity	Seq Similarity	Coverage	QMEAN Z-Scores	MolProbity Score
**N/A**	N/A	99.04%	0.61	0.89	-4.03	1.85
**rs1684873120**	M24V	98.72%	0.61	0.89	-3.94	1.88
**rs923351521**	R30L	98.72%	0.61	0.89	-3.94	1.88
**rs1684872213**	N35T	98.72%	0.61	0.89	-3.93	1.88
**rs1267066087**	I44V	98.72%	0.61	0.89	-3.88	1.83

**Table 11:** Structural validation of non-harmful nsSNP-containing proteins provided by the SWISS-MODEL web server.

rsIDs	SNPs	Ramachandran Favoured	ERRAT	PROCHECK
**N/A**	N/A	96.72%	95.1941	Errors: 3, Warning: 2, Pass: 3
**rs1684873120**	M24V	96.72%	95.7486	Errors: 1, Warning: 4, Pass: 3
**rs923351521**	R30L	96.72%	95.3789	Errors: 3, Warning: 2, Pass: 3
**rs1684872213**	N35T	96.72%	96.1326	Errors: 3, Warning: 2, Pass: 3
**rs1267066087**	I44V	96.55%	95.1941	Errors: 3, Warning: 2, Pass: 3

Finally, we did molecular docking of these proteins with CXCR-modulator-1 using the PyRx software with the same conditions (grid box dimensions: 35×35×35 Å, orientation: X:15, Y:-7.5, and Z:78). Notably, none of the mutations affected the binding affinity, with all four mutated proteins and the wild-type protein showing similar binding affinities (~ -9.1).

### Molecular dynamic simulation

**Fig 9:**
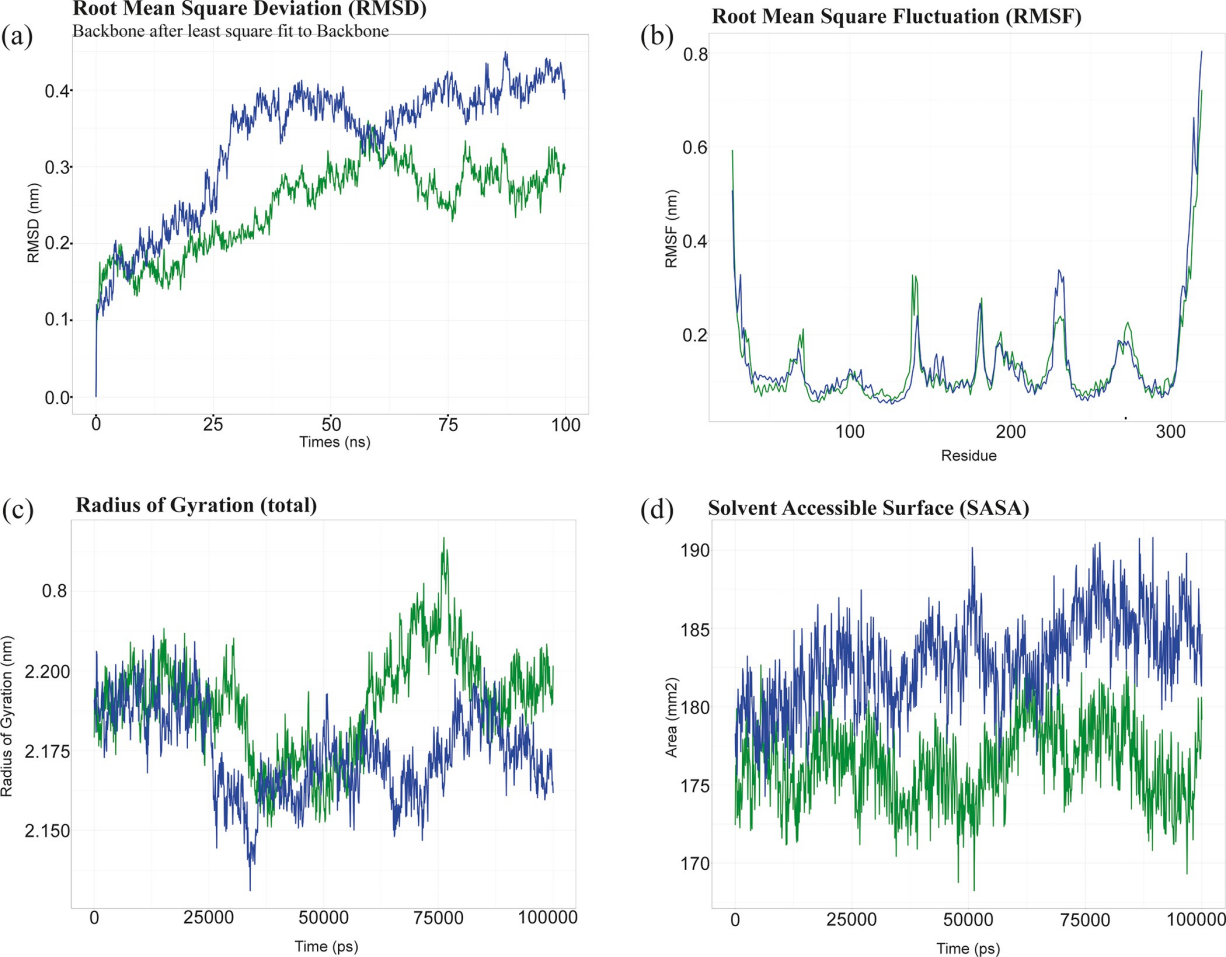
Molecular dynamics simulation. The MD simulation results show key structural properties for both the wild type (depicted in green) and the mutant (depicted in blue). (a) RMSD of the wild type and the mutant, with time in nanoseconds (ns) on the X-axis and the RMSD values in nanometers (nm) on the Y-axis represent. (b) RMSF of the wild type and the mutant are compared, using amino acid residues on the X-axis and the RMSF values in nm on the Y-axis. (c) The radius of Gyration of the wild type and the mutant, representing the time in picoseconds (ps) on the X-axis and the area in squire nanometer (nm^2^) on the Y-axis. (d) The SASA of the wild type and the mutant, with time in picoseconds (ps) on the and the SASA values in nm on the Y-axis.

To assess the stability of the systems, we employed the Root Mean Square Deviation (RMSD) calculations for wild-type and the mutant protein with H113P mutation, which are indicative of variation in protein structural integrity. Variations in RMSD value are correlated with protein structural changes. Since the beginning of the simulation, the RMSD values for the wild type and the mutant were different from each other. For the majority of the simulation, the mutant’s RMSD profile remained greater than that of the wild type (Fig 9A). Furthermore, the regional flexibility of the protein was evaluated using the Root Mean Square Fluctuation (RMSF) method. Elevated RMSF values signify increased flexibility at specific amino acid sites. Our analysis revealed varying degrees of flexibility in distinct regions between the wild-type and mutant proteins. Notably, the mutant exhibited more flexibility around the 220^th^ residue compared to the wild-type, whereas the region around the 150^th^ residue demonstrated increased flexibility in the wild-type (Fig 9B). The degree of compactness within the protein structures was measured using the radius of gyration. Comparative analysis of the mutant and the wild-type proteins revealed differences in their compactness. Notably, the mutant exhibited more compact conformations relative to the wild-type (Fig 9C). In MD simulations, the Solvent Accessible Surface Area (SASA) parameter was employed as a predictive tool to forecast the stability of the hydrophobic cores within the protein. Elevated SASA scores were indicative of an increased risk of protein destabilization, primarily attributed to increased solvent accessibility. Throughout the simulation, the mutant protein’s SASA value remained significantly higher than that of the wild-type, indicating a greater risk of solvent-induced disruption (Fig 9D).

## Discussion

The genomic databases serve as invaluable resources for generating a wealth of information through the application of different bioinformatics tools. Additionally, the utilization of bioinformatics can help in saving time and other resources. Because in vitro experiments are laborious, time and resource-consuming with no assurance of successful results. A comprehensive characterization of non-synonymous SNPs in genes associated with the specific disease is used to predict the genetic relationship between the disease and the SNPs. This is because it has been demonstrated that the SNPs are responsible for genotypic and phenotypic variations [63, 64]. This study was conducted to identify and characterize the most important nsSNPs within the CXCR4 gene. These findings may be useful for future research about disorders associated with the CXCR4 gene or its protein and may facilitate the development of novel therapeutics.

CXCR4 and its ligand stromal cell-derived factor-1 (SDF-1; also called CXCL12) are required for normal fetal development. Notably, SDF-1 plays a protective role against HIV-1 infection, while CXCR4 is a key co-receptor for T-tropic human immunodeficiency virus (HIV-1) strains. Additionally, SDF-1 and CXCR4 play pivotal roles in regulating cancer cell migration and dissemination. CXCR4 is involved in various biological processes, including HIV-1 virus receptor activity, molecular transduction, immune system process, muscle function, circulatory system regulation, cell proliferation, motility and differentiation, etc. CXCR4 is mainly studied in the HIV-1 virus fusion process, where it works as a co-receptor. The interaction between CXCR4 and, as well as the potential inhibitory compounds, can be influenced by missense SNPs within the gene. There are 2 domains in the protein–N-terminal chemokine receptor (comprising amino acid residues 6–37) and seven transmembrane domains (comprising amino acid residues 55–302), a characteristic of the rhodopsin family. Most chemokine receptors have ligand-binding domains in their N terminal region, and in the case of CXCR4, SDF-1 binds to the N terminal domain. Furthermore, Including CXCR4 exhibits physical interactions with various molecules such as CD164, CXCL14, TLR2, CCR5, etc., and demonstrates genetic interaction with the PSIP1 gene.

This study employed a comprehensive approach involving seven distinct methods to filter the missense SNPs, optimizing the utilization of various algorithms and enhancing the filtration precision. The SNPs were examined based on their predicted impact on protein function, stability, interaction with ligands, and association with diseases. Initially, five different tools, namely SIFT, Polyphen2.0, PROVEAN, PANTHER, and SNAP2.0, were employed identify the most deleterious SNPs, followed by utilization of two additional tools, PhD-SNP, SNPs & GO, to predict disease-related SNPs Out of a total of 264 non-synonymous SNPs, the 7 tools collectively filtered out 23 SNP, all of which are situated within functional domains. Mutations occurring in the chemokine receptor domain have the potential to disrupt the receptor-ligand interaction, thereby affecting receptor activity. Conversely, mutations in the transmembrane domain can cause the loss of protein function.

After finalizing the selection of the 23 nsSNPs, their impact on protein stability was systematically examined through MUpro and I-mutant3.0. The effect of substitution on decreasing protein stability suggests a potential impact of SNPs on proteins. Protein stability is intimately correlated with the Gibbs free energy. Protein stability decreases when the value is <0. Notably, a significant proportion of these SNPs, specifically 21 and 18 out of the 21 nsSNPs, exhibited a pronounced decrease in protein stability when analyzed with both MUpro and I-mutant 3.0 methodologies, respectively. The position of the amino acids within protein molecules is very crucial; mutations located in exposed regions may disrupt protein-protein interaction, while mutations in buried amino acids may lead to structural and functional alterations. These effects become severe when the mutant residues are in conserved regions. To assess the conservation and positioning of the selected amino acids, Consurf and Netsurf2.0 webservers were employed. Moreover, the Musitedeep webserver was utilized to predict the sites for glycosylation, palmitoylation, phosphorylation, and hydroxylation for wild-type and mutant residues in post-translational modification (PTM). Intriguingly, 2 extra amino acids showed the PTM (K38 and Q314) after mutation, with the remaining amino acid residues displaying distinct modifications after mutation. Following mutation, specific amino acid residues exhibited distinct post-translational modifications: Asparagine underwent glycosylation, Serine got phosphorylation and O-linked glycosylation, Arginine experienced methylation, Cysteine underwent palmitoylation, Proline was subjected to hydroxylation, Tyrosine received phosphorylation. In contrast, Aspartic acid, Leucine, Glycine, Valine, and Methionine remained unaltered, without any modification in PTM. Prediction from MutPred2.0 showed that some mutations could result in the loss of critical protein features, including loss of catalytic sites, allosteric sites, GPI-anchor amidation, and structural elements such as helices, strands, and disulfide linkages, thereby potentially compromising the overall functions of the protein. Besides, some mutations were found to induce gains in protein features, such as allosteric sites, solvent accessibility, intrinsic disorder, and structural elements like loops, helices, and strands, leading to functional changes or losses. Additionally, HOPE predicted that substitutions involving bigger residues could make it difficult to fit into the protein and thus may disrupt the conformation, while substitutions with smaller residues might hinder protein folding, ultimately causing structural destabilization.

After knowing the positions of the amino acid residues in the helices, strands, and loops of the protein using SOPMA and the clustering of mutations in the Mutation 3D web server, homology modeling was conducted in a templet-based method in ’SWISS-’MODEL’ considering "3odu.1.A" as a template. These variants were found to be clustered in 7 different groups. Subsequently, to compare the differences in the 3D protein structures between the wild-type and mutant proteins, the RMSD and TM align values were calculated using the wild-type structure as the reference. A larger RMSD value signifies structural disparity between the wild-type and mutant protein, while TM align values >0.5 and 1 indicate structural dissimilarity. Three substitutions, namely G55V, H79P, L80P, H113P, and P299L, were selected for further analysis due to their higher RMSD and lower TM values relative to other selected residues. In addition to these three substitutions, P299L was also selected because of its distinctive effect on the protein structure, resulting in the loss of disulfide bond, the acquisition of Intrinsic disorder, the addition of pyrrolidone carboxylic acid at Q314, and alterations in disordered interfaces, and transmembrane properties. All five substituted amino acids were further subjected to in-depth analysis using the BIOVIA discovery studio visualizer.

The ultimate effect of the mutations on the structure and function of the protein can be best analyzed by docking the protein with its ligand. The natural ligand of CXCR4 is CXCL12. However, protein-protein docking can be more complicated and often yields inaccurate results, especially in cases where the binding sites or domains are poorly characterized. However, performing protein-ligand docking with a small chemical molecule as the ligand is easier and more convenient. In such cases, the protein serves as the macromolecule and the small chemical compound acts as the ligand, enabling a high degree of accuracy and precision. This is the reason why we started looking for small ligand molecules that could interact with CXCR4. Our investigation led us to CXCR4-modulator 1, a ligand commonly used in therapeutic research for its interaction with CXCR4. The docking result showed that the mutant variants of the protein obtained from our study exhibited either similar or enhanced affinity and binding energy when interacting with the ligand. Therefore, the result suggests that the mutant protein maintains, in some cases augments, its affinity for the ligand than the wild-type structure. Since the ligand is used as a drug to inhibit the function of CXCR4, and the mutant CXCR4 binds even better with the ligand, it indicates that the drugs designed as analogs of CXCR4-modulator 1 would inhibit CXCR4 with better efficacy in people harboring the mutant variants of CXCR4 protein, especially those featuring the H79 variant. However, further research in a laboratory setting is required to conclusively validate the favorable effects of these mutant proteins on these drugs.

It is important to note that the H113P SNP is positioned within the binding site of the CXCR4 protein. Along with Key residues of CXCR4 that are involved in the binding of a small ligand, like a drug are Trp94, Asp 97, Asp171, Arg183, Asp187, Arg188, Tyr190, Asp 193, Asp262, Glu288 and His113. His113 is proximal to this binding region, perhaps making the mutation from Histidine to Proline at position 113 significantly impactful on the binding affinity of the protein with small ligands. Therefore, this provides insights into the broader implications for CXCR4 function in cellular processes and potential therapeutic targeting.

Additionally, molecular dynamics (MD) simulations of the protein-ligand complex for both the wild-type and H113P mutant proteins revealed significant differences in key structural parameters, including RMSD, RMSF, radius of gyration, and SASA profiles. The simulations were conducted over a 100 ns timeframe, consistent with previously established studies [65–68]. These variations in structural dynamics strongly indicate that the H113P mutation is likely to disrupt the normal function of the CXCR4 protein, potentially leading to alterations in its stability, flexibility, and ligand-binding behavior.

SNPs located in the non-coding regions of a gene can have significant functional implications, influencing transcription factor binding, chromatin accessibility, and alternative splicing, among other processes. The RegulomeDB web server provides detailed insights into the functional impact of non-coding SNPs, including information on overlapped transcription factor motifs, matched footprints, and chromatin states. The ranking system used by RegulomeDB reflects the depth of available data on each SNP. In our analysis, the majority of non-coding SNPs in the CXCR4 gene were classified as rank 4. This indicates that most of these SNPs likely affect transcription factor binding sites and are also supported by chromatin accessibility assays, suggesting their potential regulatory role in gene expression.

In silico bioinformatics tools were employed for all predictions, making it crucial to evaluate their sensitivity and specificity to validate the results. We filtered non-damaging nsSNPs and assessed their impact on the protein’s structure and function under similar conditions. For this, four nsSNPs (M24V, R30L, N35T, and I44V) located at the N-terminal were identified as non-deleterious and non-disease-causing and examined their impact on the structure and function of the CXCR4. These nsSNPs did not affect the affinity of the protein for the selected drug, indicating that the bioinformatics tools used in this study provided practical and reliable results.

Our study provides valuable insights into the biological impact of SNPs in the CXCR4 gene. Although the direct implications of these genetic abnormalities in diseases are beyond the scope of this work, our findings suggest that the identified SNPs can significantly alter CXCR4 function and its interactions with other biomolecules. Given that CXCR4 is widely expressed in immune cells and hematopoietic stem cells, these disruptions can have far-reaching consequences. From a network biology perspective, the failure of a critical node (such as CXCR4) to interact with its associated nodes can lead to various cellular abnormalities. Our molecular docking and molecular dynamics simulation results strongly indicate that these SNPs may destabilize cellular behavior in CXCR4-expressing cells, potentially contributing to the onset or progression of multiple diseases. Future studies are warranted to investigate specific metabolic and physiological changes that may arise due to these SNPs, providing a clearer understanding of their role in disease mechanisms.

However, while these computational predictions provide valuable insights, they require experimental validation. This study identified important mutations that may impact the CXCR4 protein, but laboratory-based case-control studies are essential for confirmation. Conducting site-directed mutagenesis to introduce these mutations and comparing the effects on mutant and wild-type proteins was beyond the scope of this research. Future studies should focus on validating these findings through laboratory experiments. Additionally, since SNP effects are often population-specific, it is crucial to consider population bias during experimental investigations. Additionally, as SNP effects can vary across different populations, it is critical to account for potential population biases in future studies. Certain SNPs are known to be specific to populations based on race, ethnicity, or gender, which may predispose certain groups to particular diseases. For example, previous studies have examined specific SNPs in populations such as the Chinese Han, African, and Taiwanese populations, finding associations with various diseases [69–71]. Our study provides a list of deleterious SNPs, but further experimental validation using a representative, unbiased population sample is essential.

One of the challenges we encountered in this study was the frequent updates of computational tools and databases. As new versions are released, they often include updated information that can impact the results. To ensure the reliability of our findings, we repeated several analyses with the latest versions available during our study. However, there remains the possibility that our article may not reflect the most recent updates at the time of publication. Despite this, we believe that the comprehensive methodology, detailed results, and thorough discussion provided in this manuscript offer an easy-to-follow workflow that enhances the reproducibility of the work.

Several previous studies on CXCR4 gene reported multiple mutations that are associated with certain diseases such as WHIM syndrome [72] and Waldenström’s Macroglobulinemia [73]. Our study provides a comprehensive characterization of all possible SNPs within the gene, facilitating the assessment of its association with other diseases involving this protein. Future case reports can be correlated with our findings to better understand the clinical implications of these genetic variants.

## Supporting information

S1 Fig The percentages of the found and categorized non-coding SNPs provided by the RegulomeDB webserver. SNPs in the (a) 3’ UTR region are categorized into 4 ranks - 2a (1%), 2b (18%), 3a (3%), and 4 (78%); in (b) intron region are categorized into 8 ranks– 1b (1%), 1f (0%), 2a (11%), 2b (14%), 2c (0%), 3a (2%), 4 (71%), and 5 (1%); and the SNPs in (c) 5’ UTR region are categorized into 2 ranks– 2b (10%) and 4 (90%).
(PDF)

S1 Table Rank of SNPs in the non-coding regions provided by RegulomeDB webserver. (PDF)

S2 Table Information holds by the ranks in RegulomeDB webserver. (PDF)
