# Supplementary material for: Comprehensive characterization of high-risk coding and non-coding single nucleotide polymorphisms of human CXCR4 gene
Source: PLoS One. 2024 Dec 23;19(12):e0312733. doi: 10.1371/journal.pone.0312733 (PMC11665994; doi:10.1371/journal.pone.0312733)
Supplement: S1 Fig — SNPs in the (a) 3’ UTR region are categorized into 4 ranks - 2a (1%), 2b (18%), 3a (3%), and 4 (78%); in (b) intron region are categorized into 8 ranks– 1b (1%), 1f (0%), 2a (11%), 2b (14%), 2c (0%), 3a (2%), 4 (71%), and 5 (1%); and the SNPs in (c) 5’ UTR region are categorized into 2 ranks– 2b (10%) and 4 (90%). (PDF) [file pone.0312733.s001.pdf]

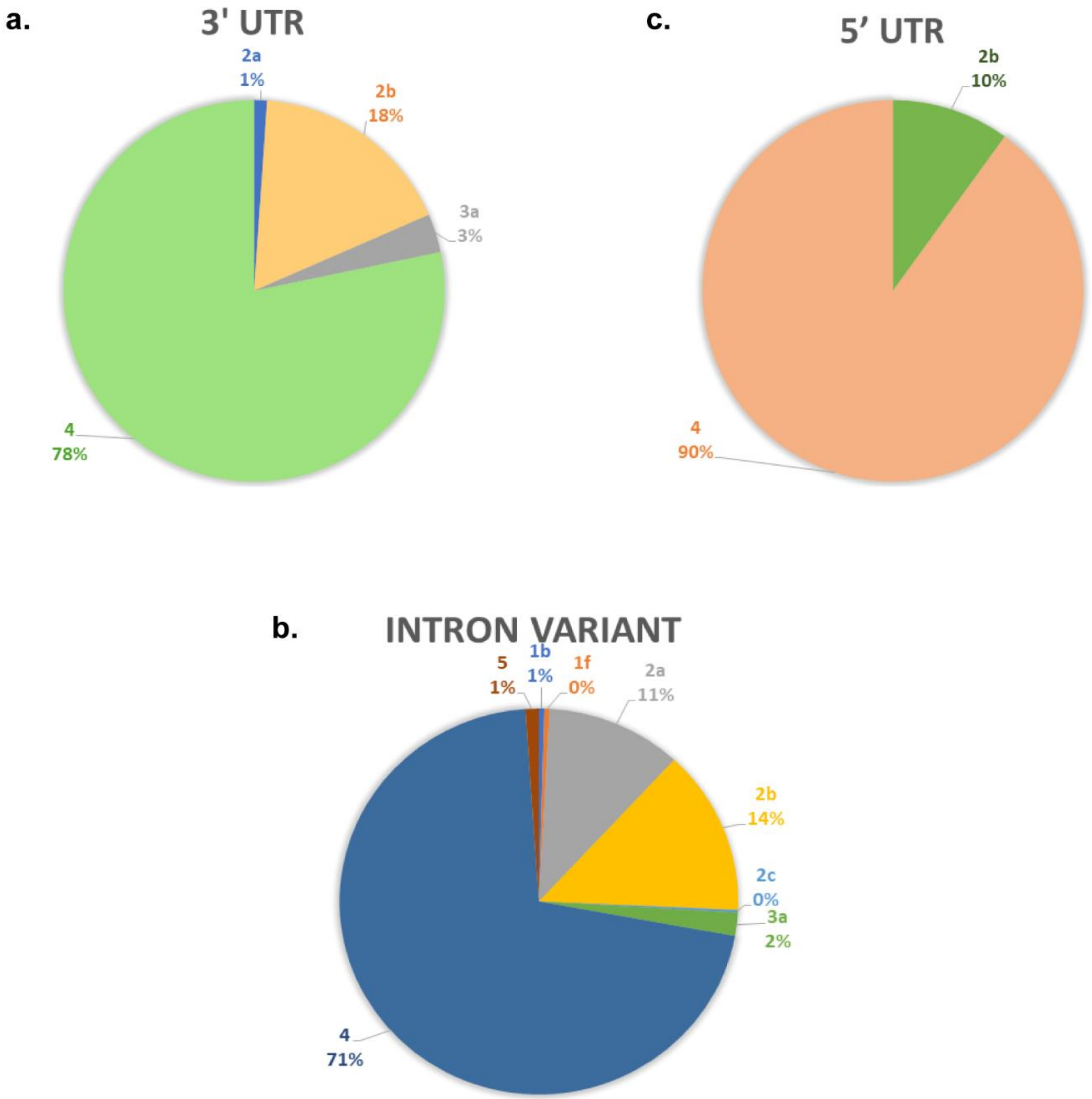

S1 Fig: The percentages of the found and categorized non-coding SNPs provided by the RegulomeDB webserver. SNPs in the (a) 3' UTR region are categorized into 4 ranks - 2a (1%), 2b (18%), 3a (3%), and 4 (78%); in (b) intron region are categorized into 8 ranks – 1b (1%), 1f (0%), 2a (11%), 2b (14%), 2c (0%), 3a (2%), 4 (71%), and 5 (1%); and the SNPs in (c) 5' UTR region are categorized into 2 ranks – 2b (10%) and 4 (90%).
