# Supplementary material for: Comprehensive characterization of high-risk coding and non-coding single nucleotide polymorphisms of human CXCR4 gene
Source: PLoS One. 2024 Dec 23;19(12):e0312733. doi: 10.1371/journal.pone.0312733 (PMC11665994; doi:10.1371/journal.pone.0312733)
Supplement: S1 Table — (PDF) [file pone.0312733.s002.pdf]

S1 Table: Rank of SNPs in the non-coding regions provided by RegulomeDB webserver.

Regions	Number of SNPs		Ranks
	From Ensembl	Found in RegulomeDB	
**3' UTR**	156	91	2a (1), 2b (16), 3a (3), 4 (72)
**Intron Variant**	1063	463	1b (2), 1f (2), 2a (51), 2b (64), 2c (1), 3a (9), 4 (329), 5 (5)
**5' UTR**	47	30	2b (3), 4 (27)
