# Supplementary material for: Comprehensive characterization of high-risk coding and non-coding single nucleotide polymorphisms of human CXCR4 gene
Source: PLoS One. 2024 Dec 23;19(12):e0312733. doi: 10.1371/journal.pone.0312733 (PMC11665994; doi:10.1371/journal.pone.0312733)
Supplement: S2 Table — (PDF) [file pone.0312733.s003.pdf]

S2 Table: Information holds by the ranks in RegulomeDB webserver.

Ranks	Supporting Data
**1a**	eQTL/caQTL, TF binding, matched TF motif, matched Footprint, chromatin accessibility peak
**1b**	eQTL/caQTL, TF binding, any motif, Footprint, chromatin accessibility peak
**1c**	eQTL/caQTL, TF binding, matched TF motif, chromatin accessibility peak
**1d**	eQTL/caQTL, TF binding, any motif, chromatin accessibility peak
**1e**	eQTL/caQTL, TF binding, matched TF motif
**1f**	eQTL/caQTL, TF binding or chromatin accessibility peak
**2a**	TF binding, matched TF motif, matched Footprint, chromatin accessibility peak
**2b**	TF binding, any motif, Footprint, chromatin accessibility peak
**2c**	TF binding, matched TF motif, chromatin accessibility peak
**3a**	TF binding, any motif, chromatin accessibility peak
**3b**	TF binding + matched TF motif
**4**	TF binding, chromatin accessibility peak
**5**	TF binding or chromatin accessibility peak
**6**	Motif hit
**7**	Other
